# Inhibition of Bruton’s tyrosine kinase with PD-1 blockade modulates T cell activation in solid tumors

**DOI:** 10.1172/jci.insight.169927

**Published:** 2024-11-08

**Authors:** Emily Schwarz, Brooke Benner, Robert Wesolowski, Dionisia Quiroga, Logan Good, Steven H. Sun, Himanshu Savardekar, Jianying Li, Kyeong Joo Jung, Megan C. Duggan, Gabriella Lapurga, Jami Shaffer, Luke Scarberry, Bhavana Konda, Claire Verschraegen, Kari Kendra, Manisha Shah, Robert Rupert, Paul Monk, Hiral A. Shah, Anne M. Noonan, Kristin Bixel, John Hays, Lai Wei, Xueliang Pan, Gregory Behbehani, Yang Hu, Olivier Elemento, Dongjun Chung, Gang Xin, Bradley W. Blaser, William E. Carson

**Affiliations:** 1 Comprehensive Cancer Center,; 2 Division of Medical Oncology, Department of Internal Medicine,; 3 Division of Surgical Oncology, Department of Surgery,; 4 Department of Biomedical Informatics, College of Medicine,; 5 Division of Gynecologic Oncology, Department of Obstetrics and Gynecology,; 6 Center for Biostatistics, and; 7 Division of Hematology, Department of Internal Medicine, The Ohio State University, Columbus, Ohio, USA.; 8 Caryl and Israel Englander Institute for Precision Medicine, Weill Cornell Medicine, Cornell University, New York, New York, USA.

**Keywords:** Clinical trials, Immunology, Cancer immunotherapy, Cellular immune response, T cells

## Abstract

**BACKGROUND:**

Inhibition of Bruton’s tyrosine kinase with ibrutinib blocks the function of myeloid-derived suppressor cells (MDSC). The combination of ibrutinib and nivolumab was tested in patients with metastatic solid tumors.

**METHODS:**

Sixteen patients received ibrutinib 420 mg p.o. daily with nivolumab 240 mg i.v. on days 1 and 15 of a 28-day cycle. The effect of ibrutinib and nivolumab on MDSC, the immune profile, and cytokine levels were measured. Single-cell RNA-Seq and T cell receptor sequencing of immune cells was performed.

**RESULTS:**

Common adverse events were fatigue and anorexia. Four patients had partial responses and 4 had stable disease at 3 months (average 6.5 months, range 3.5–14.6). Median overall survival (OS) was 10.8 months. Seven days of Bruton’s tyrosine kinase (BTK) inhibition significantly increased the proportion of monocytic-MDSC (M-MDSC) and significantly decreased chemokines associated with MDSC recruitment and accumulation (CCL2, CCL3, CCL4, CCL13). Single-cell RNA-Seq revealed ibrutinib-induced downregulation of genes associated with MDSC-suppressive function (*TIMP1*, *CXCL8*, *VEGFA*, *HIF1A*), reduced MDSC interactions with exhausted CD8^+^ T cells, and decreased TCR repertoire diversity. The addition of nivolumab significantly increased circulating NK and CD8^+^ T cells and increased CD8^+^ T cell proliferation. Exploratory analyses suggest that MDSC and T cell gene expression and TCR repertoire diversity were differentially affected by BTK inhibition according to patient response.

**CONCLUSION:**

Ibrutinib and nivolumab were well tolerated and affected MDSC and T cell function in patients with solid metastatic tumors.

**TRIAL REGISTRATION:**

ClinicalTrials.gov NCT03525925.

**FUNDING:**

NIH; National Cancer Institute Cancer; National Center for Advancing Translational Sciences; Pelotonia.

## Abstract 2

A pilot study of Bruton’s tyrosine kinase inhibitor ibrutinib alone and in combination with PD-1 inhibitor nivolumab in patients with metastatic solid tumors.

## Introduction

Immune checkpoint inhibitors (ICIs) are antibodies that block T cell inhibitory signals. They are the standard of care in many cancers (1). However, not all patients respond, and a substantial proportion experience only partial or temporary benefit. Understanding the mechanisms underlying resistance to ICIs is an area of intense investigation. One potential source of ICI resistance are immune cells that can suppress antitumor immune responses.

Myeloid-derived suppressor cells (MDSC) are an immunosuppressive population of immature myeloid cells. In cancer, we and others have shown that MDSC abnormally expand and migrate to tumor/lymphoid regions where they negatively affect immune cells via production of reactive oxygen species and inhibitory cytokines such as TGF-β and IL-10 (2, 3). MDSC levels correlate with tumor burden and have prognostic value in many solid tumors (4–7). Murine models have shown that MDSC depletion or inhibition results in reduced tumor growth and improved antitumor immunity (2, 8).

Ibrutinib is an irreversible inhibitor of Bruton’s tyrosine kinase (BTK) that has revolutionized treatment of chronic lymphocytic leukemia (CLL) and is FDA approved for several hematologic indications (9). BTK is important to B cell function, and its inhibition can impair their migration, adhesion, and survival (10). One potential barrier to ICI efficacy may be the effects of MDSC on the tumor microenvironment (8, 11, 12). Our group has shown that MDSC express BTK, and BTK signaling plays a role in MDSC development and activation (13, 14). Specifically, ibrutinib blocked MDSC function and reversed their inhibition of T cells and NK cells (2, 10). Ibrutinib inhibited in vitro generation of human MDSC, and pretreatment of MDSC with ibrutinib significantly impaired nitric oxide production and migration. Ibrutinib treatment of tumor-bearing mice reduced splenic and tumor MDSC frequencies and enhanced the efficacy of anti–programmed cell death ligand 1 (PD-L1) therapy (15, 16).

Nivolumab is a human monoclonal IgG4κ antibody against programmed cell death protein 1 (PD-1) that is FDA approved for multiple malignancies, including melanoma, non–small cell lung carcinoma, and Hodgkin’s lymphoma (17). Nivolumab blocks the interaction of PD-1 with its ligand PD-L1, resulting in release of T cell inhibition and restoration of T cell–mediated antitumor responses (1). The overall response rate to nivolumab ranges from 20% to 40%, and thus, efforts to improve its efficacy are needed.

The ability of ibrutinib to inhibit MDSC and thereby improve antitumor immune responses suggests that it could increase the efficacy of ICI. This study was conducted to evaluate the clinical and immunologic effects of adding ibrutinib to nivolumab in patients with metastatic solid tumors.

## Results

### Patient characteristics.

**Figure 1:**
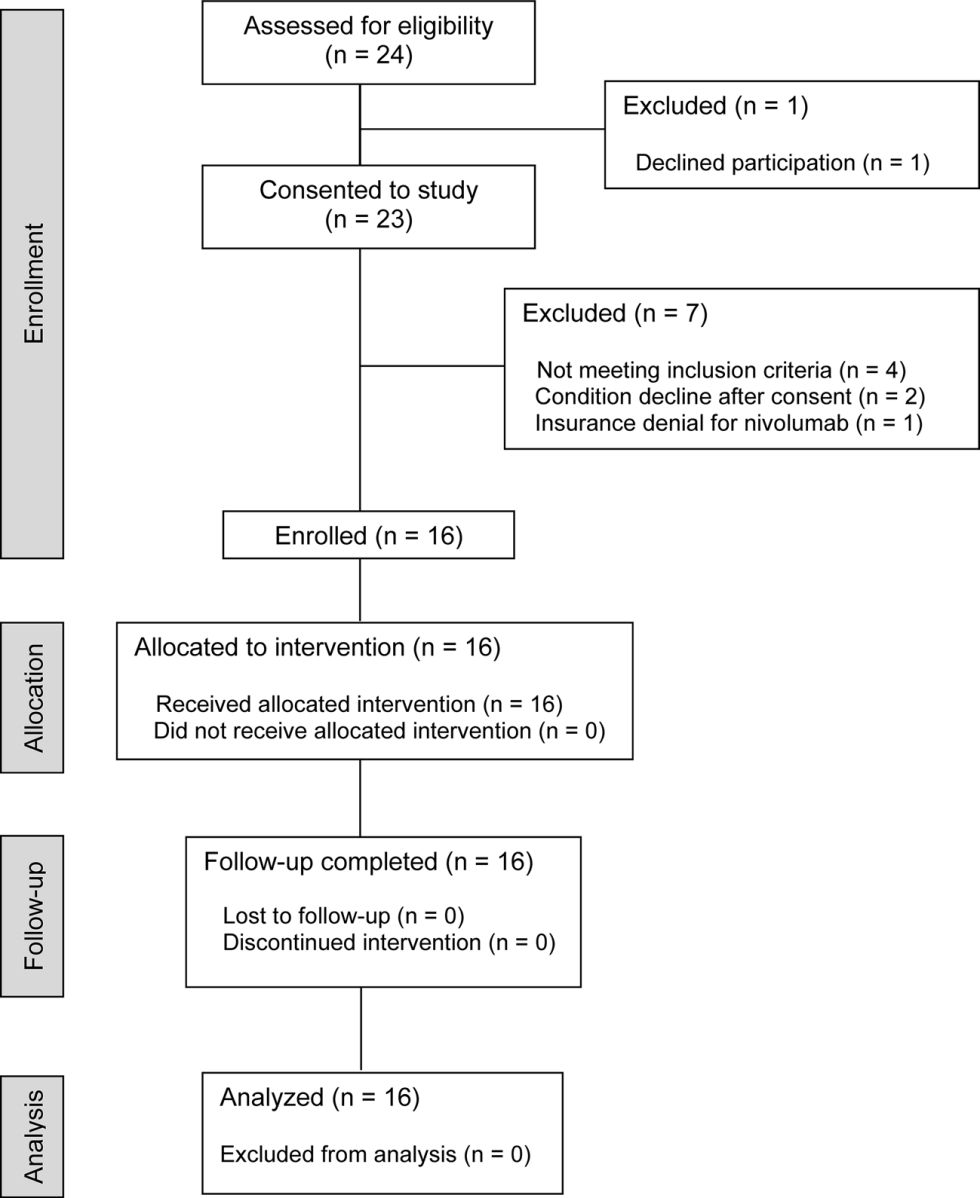
CONSORT diagram. Sixteen patients with metastatic solid tumors were enrolled in the pilot phase I study. All patients were allocated to the same treatment intervention (ibrutinib 420 mg p.o. daily with nivolumab 240 mg i.v. on days 1 and 15 of a 28-day cycle).

A pilot study of ibrutinib with nivolumab was conducted in 16 patients with metastatic solid tumors (Figure 1). The treatment schema is displayed in Supplemental Table 1 (supplemental material available online with this article; https://doi.org/10.1172/jci.insight.169927DS1). Ibrutinib dosing was started 7 days prior (C1D–7) to the start of nivolumab therapy (C1D1) and given in combination with nivolumab until day 8 of cycle 1 (C1D8). Peripheral blood was collected prior to initiation of ibrutinib (C1D–7) and prior to drug administration on days 1 (C1D1) and 8 (C1D8) of cycle 1, day 1 of cycle 2 (C2D1), and at the time of disease progression. Patient demographics and characteristics are summarized in Supplemental Table 2. The median age was 60.5 years (range 31–81). The majority of patients were White (88%, *n* = 14), 6% were African American (*n* = 1), and 6% declined to answer (*n* = 1). In total, 56% percent of patients were male (*n* = 9) and 44% were female (*n* = 7). The most frequent cancer types were melanoma (25%), neuroendocrine (19%), thyroid (13%), and pancreatic adenocarcinoma (13%). The majority of patients had an Eastern Cooperative Oncology Group (ECOG) performance score of 0 or 1 (94%) and had received prior systemic therapy (75%). Five patients (31%) were treatment naive, and 25% of patients had 1 line of prior therapy. Seven patients (44%) had received 3 or more prior treatment regimens. No patients had received prior ICI therapy.

### Safety.

All patients received at least 7 days of ibrutinib (average 13 days, range 7–15 days) and at least 1 dose of nivolumab. There were no grade 4 or 5 events. There were no dose reductions; however, treatment was delayed in 4 patients due to grade 3 AEs (maculopapular rash, nausea, lung infection) and grade 2 atrial fibrillation (Supplemental Table 3). The most common adverse events (AEs) were fatigue (31%), anorexia (31%), and maculopapular rash (25%). Grade 1 fatigue occurred in 4 patients, and grade 2 fatigue occurred in 1 patient. Grade 1 anorexia occurred in 3 patients, and grade 2 anorexia occurred in 2 patients. Grade 1 maculopapular rash occurred in 2 patients, and grade 3 maculopapular rash occurred in 2 patients. These toxicities were similar to other studies that employed ibrutinib/nivolumab (18–20).

### Clinical efficacy.

Tumor responses are summarized in Supplemental Table 4. Four patients (25%) experienced a partial response (PR), 4 (25%) achieved stable disease (SD) (average length 6.5 months, range 3.5–14.6 months), and the remainder had progressive disease (PD). Of the patients with PR, 3 had melanoma and 1 had a neuroendocrine tumor. Of the patients with SD, 1 had thymic carcinoma (SD lasting 3.5 months), 1 had thyroid cancer (SD lasting 4.3 months), 1 had mucinous adenocarcinoma (SD lasting 14.6 months), and 1 had neuroendocrine cancer (SD lasting 3.5 months). The clinical impression of the treating physicians was that the patients classified as having SD had clinical trajectories that were noticeably affected by the treatment and distinct from patients with disease progression. Supplemental Figure 1A summarizes the greatest reduction in tumor size in 12 patients. Four patients were not eligible for this measurement due to no follow-up imaging being completed at progression. Among all patients, median progression free survival (PFS) was 3.5 months and median overall survival (OS) was 10.8 months (Supplemental Figure 1, B and C). Treatment naive patients had a longer median PFS (9.8 months) than those who had received prior systemic therapies (2.7 months, *P* = 0.42). A swimmer plot depicting treatment durations is shown in Supplemental Figure 2.

### Levels of circulating MDSC.

**Figure 2:**
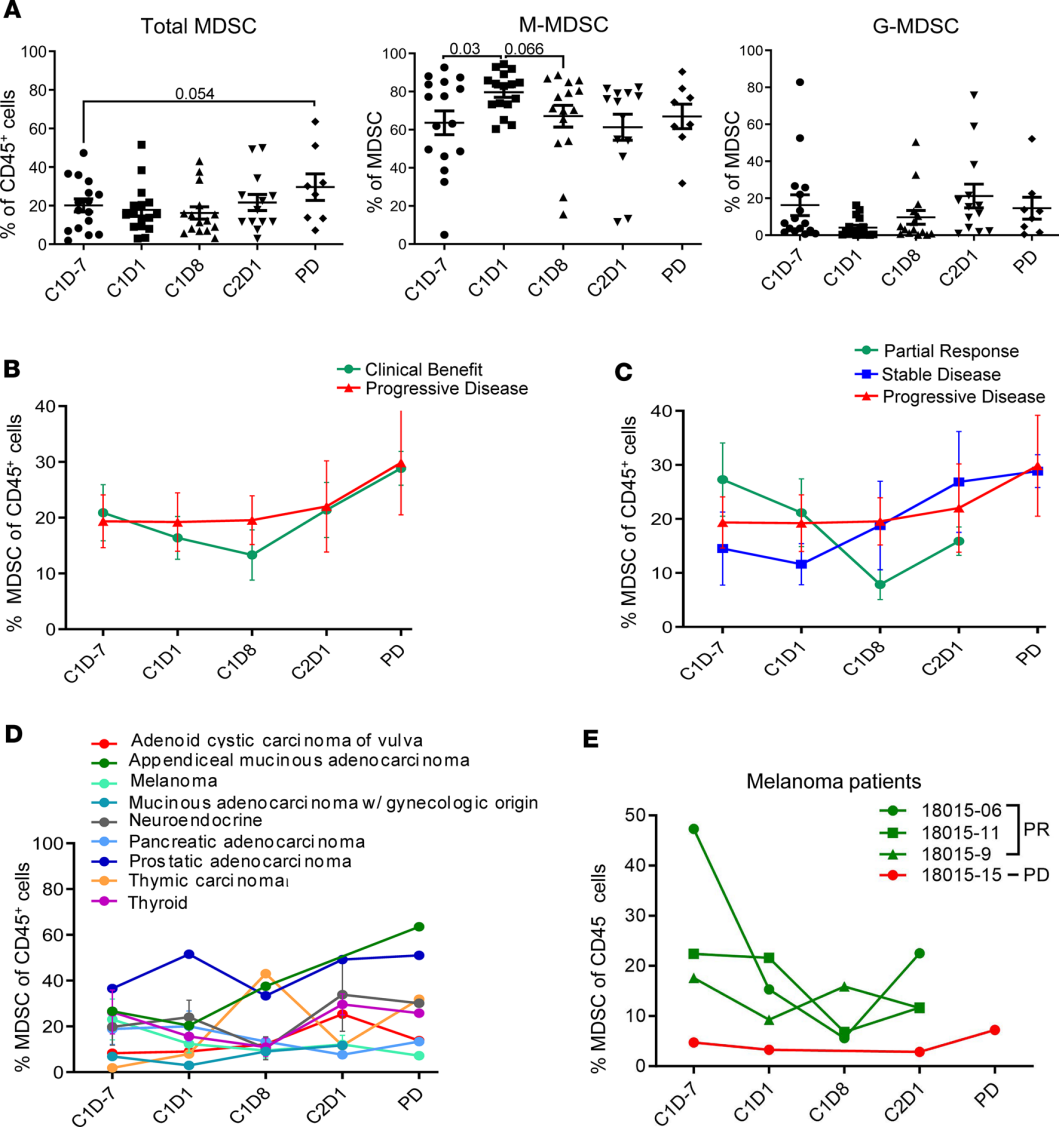
Levels of circulating myeloid-derived suppressor cells. (**A**) Circulating levels of total (CD11b^+^, CD33^+^, HLA-DR^lo/–^), monocytic (CD14^+^) or granulocytic (CD66b^+^) MDSC were measured by mass cytometry at cycle 1 day –7 (baseline), after 1 week of ibrutinib (cycle 1 day 1), cycle 1 day 8, cycle 2 day 1, and at disease progression (PD) (*n* = 16). Data represent mean ± SEM and were analyzed by paired Student’s *t* test. The *P* values were adjusted for multiple comparisons using Holm-Bonferroni method. (**B** and **C**) MDSC levels by best response for patients with clinical benefit (partial response and stable disease, *n* = 8) versus progressive disease (*n* = 8) (**B**) and patients with partial response (*n* = 4) versus stable disease (*n* = 4) versus progressive disease (*n* = 8) (**C**). Data represent mean ± SEM and were analyzed using Student’s *t* test (unpaired) in **B** and ANOVA in **C**. The *P* values were adjusted for multiple comparisons using Holm-Bonferroni. (**D**) Average MDSC levels by tumor type. (**E**) MDSC levels from patients with melanoma (*n* = 4).

It was hypothesized that BTK inhibition by ibrutinib would reduce MDSC frequency and/or function. To measure changes in the levels of circulating MDSC associated with ibrutinib and nivolumab therapy, PBMC were analyzed via mass cytometry (gating strategy in Supplemental Figure 3). The average frequency of total MDSC at baseline (C1D–7) was 20.12% ± 3.35% in all patients. MDSC levels decreased to an average of 17.80% ± 3.16% after 7 days of ibrutinib (C1D1) and to 16.23% ± 3.16% by day 8 of course 1 (C1D8, *P* = 1). MDSC then increased to 21.62% ± 4.17% by C2D1, although this increase was not significant (*P* = 1). Levels of MDSC increased further to 29.59% ± 6.87% at the time of disease progression, which were markedly higher than baseline levels (*P* = 0.054; Figure 2A). The proportions of monocytic-MDSC (M-MDSC) and granulocytic-MDSC (G-MDSC) subsets were also examined (21). The proportion of M-MDSC significantly increased from 63.63% ± 6.23% at C1D–7 to 79.64% ± 2.70% at C1D1 (*P* = 0.033) and then subsequently decreased to 67.09% ± 5.70% by C1D8 (*P* = 0.066, Figure 2A). Conversely, the proportion of G-MDSC decreased on average from 16.28% ± 5.63% at C1D–7 to 4.125% ± 1.36% at C1D1 (*P* = 0.10). G-MDSC then increased to an average of 14.64% ± 5.93% at disease progression.

An additional analysis of total MDSC levels according to clinical response is provided in Figure 2B. Patients were classified into 1 of 2 response groups, with the “clinical benefit” group representing patients who experienced a PR or SD as their best response and the second group being patients who experienced PD. No significant differences in MDSC levels were found between these 2 response groups, although MDSC levels at C1D8 were lower on average in patients with clinical benefit than in patients with PD (Figure 2B). MDSC levels in patients with PR, SD, and PD were also investigated (Figure 2C). Patients with PR had higher baseline MDSC levels (27.28% ± 6.76%) compared with SD (14.52% ± 6.77%) and patients with PD (19.34% ± 4.74%) and exhibited the greatest reduction in MDSC between C1D–7 and C1D8. MDSC levels at C1D8 in patients with PR were lower than those of patients with PD (7.86% ± 2.79% vs. 19.56% ± 4.39%, *P* = 0.07) although this result did not achieve significance. While patients with SD and PD had overall increases in MDSC levels between baseline and the time of disease progression, PR patient MDSC levels decreased 11.38% on average between baseline and C2D1.

Total MDSC frequency over time is summarized by tumor type in Figure 2D. Most tumor types mimicked the overall trend for MDSC presented in Figure 2A. Notably, thymic carcinoma, mucinous adenocarcinoma of gynecologic origin, and adenoid cystic carcinoma of vulva displayed lower levels of MDSC at baseline compared with other tumor types, on average. Treatment-induced changes in MDSC subsets were highest in melanoma, mucinous adenocarcinoma, and thyroid and thymic carcinoma (Supplemental Figure 4). An additional analysis of MDSC levels in the 4 patients with melanoma is presented in Figure 2E. Notably, 3 of 4 patients with PR in this study had melanoma, and these patients had higher levels of MDSC at baseline than the 1 melanoma patient with PD. However, limited sample sizes within each tumor type prevented any statistical differences in MDSC levels by disease origin.

### Changes in additional immune subsets following ibrutinib and nivolumab treatment.

**Figure 3:**
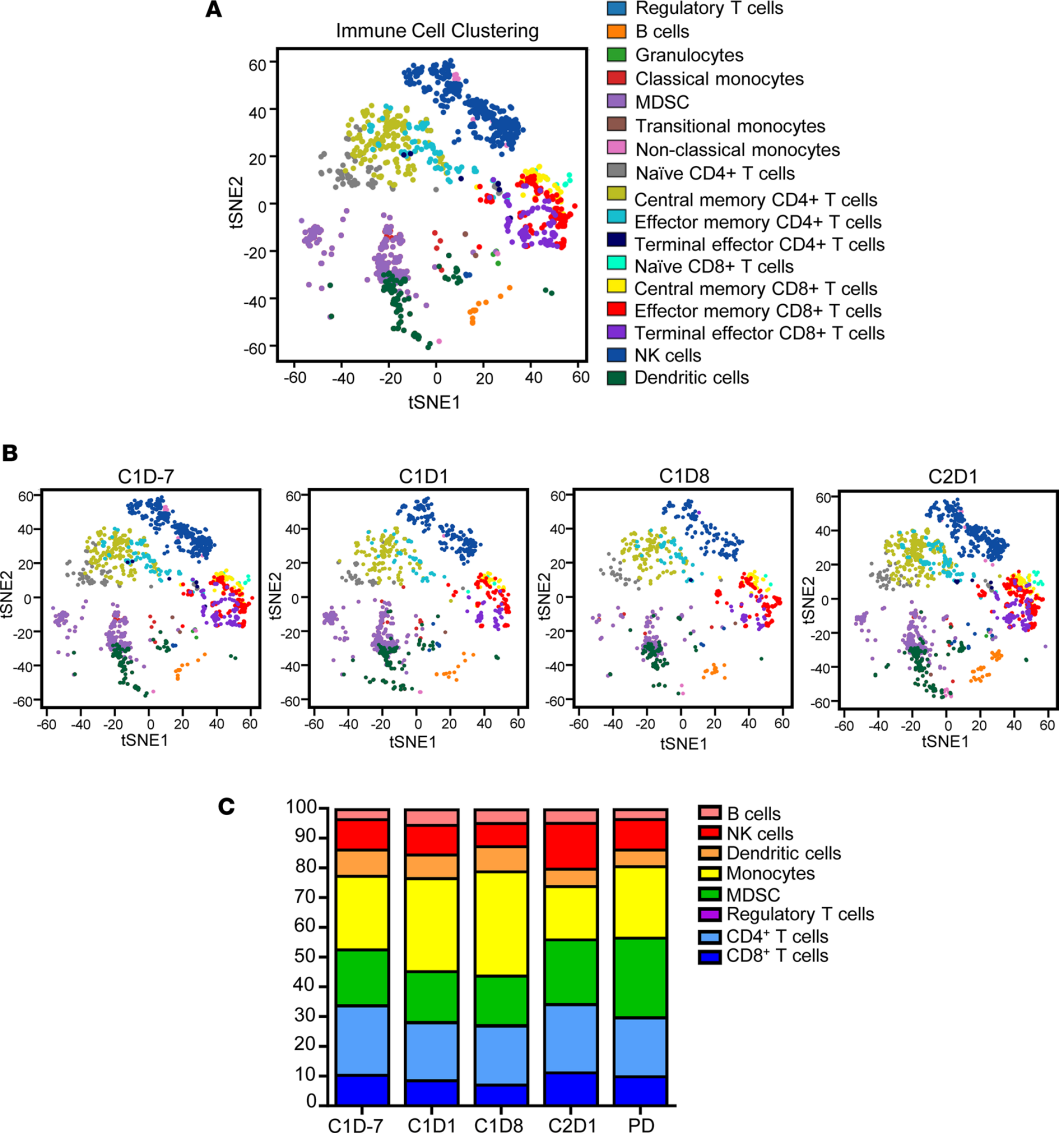
Overall immune cell profile in patients with metastatic solid cancer following treatment with ibrutinib and nivolumab. Mass cytometry analysis of patient PBMC at baseline (C1D–7), following treatment with single-agent ibrutinib (C1D1) or ibrutinib in combination with nivolumab (C1D8, C2D1) and at the time of disease progression (PD) in 16 patients with metastatic solid tumors. (**A**) Representative t-SNE plot of immune cells clustered in an unbiased manner from live/CD45^+^ cells. (**B**) Representative t-SNE plots of immune cell population clustering from 1 patient with metastatic solid disease over the course of the study. (**C**) Bar graph of mean immune cell populations in patients with metastatic solid tumors (*n* = 16) over the duration of the study.

The use of the 37-marker mass cytometry panel (Supplemental Table 5) permitted the identification of 35 immune cell populations (Supplemental Table 6) and an analysis of posttreatment changes across the immune compartment. For the study as a whole, immune subsets were identified in an unbiased manner using viSNE, a visualization tool for high-dimensional single-cell data based on t-distributed stochastic neighbor embedding (t‑SNE) (Figure 3A). Ibrutinib treatment induced notable shifts in circulating immune cell populations compared with baseline. A representative patient with PR is presented in Figure 3B and demonstrates the overall trend for MDSC changes across treatment (down at C1D8). For the entire cohort (Figure 3C), significant decreases in total CD4^+^ T cells (24.93% ± 2.50% to 20.30% ± 2.05%, *P* = 0.04) and decreases in total CD8^+^ T cells (11.51% ± 1.45% to 9.32% ± 1.19%, *P* = 0.05) were observed from C1D–7 to C1D1 (Supplemental Figure 5, A and B). No significant changes were seen in levels of Tregs, Th1-like, Th2-like, Th17-like, or γδT cells with treatment (Supplemental Figure 5C). PD-1 expression on total T cells, CD8^+^ T cells, and CD4^+^ T cells was not significantly altered by single-agent ibrutinib treatment (Supplemental Figure 5D). There was no change in levels of NK cells, total B cells, or naive B cells in response to ibrutinib alone, although memory B cells significantly increased from C1D–7 to C1D1 (0.28% ± 0.05% to 0.53% ± 0.36%, *P* = 0.004; Supplemental Figure 6A). Classical monocytes increased nonsignificantly from C1D–7 to C1D1 (26.47% ± 2.80% to 32.64% ± 2.19%, *P* = 0.14; Supplemental Figure 6B) while total, plasmacytoid, myeloid DCs (mDCs), granulocytes, and neutrophils were largely unaffected (Supplemental Figure 6, C and D). Next, the effects of combining ibrutinib and nivolumab were evaluated. Total CD4^+^ T cells (19.30% ± 2.44% to 24.77% ± 3.17%, *P* = 0.28) increased nonsignificantly (Supplemental Figure 5A), while total CD8^+^ T cells (8.13% ± 1.32% to 11.57% ± 1.75%, *P* = 0.04; Supplemental Figure 5B) and NK cells (8.66% ± 1.32% to 15.41% ± 2.24%, *P* = 0.04; Supplemental Figure 6A) increased significantly following the addition of nivolumab therapy. Classical monocytes (35.32% ± 2.80% to 22.31% ± 3.59%, *P* = 0.05), DCs (9.60% ± 1.54% to 5.79% ± 0.99%, *P* = 0.02), and mDC (6.37% ± 0.76% to 3.73% ± 0.57%, *P* = 0.003) decreased from C1D8 to C2D1 (Supplemental Figure 6, B and C). Lastly, immune populations were evaluated for differences between patients with clinical benefit and patients with PD. Patients with clinical benefit had significantly higher baseline levels of total NK cells compared with patients with PD (*P* = 0.046). No differences were found between response groups for CD56^bright^, CD56^dim^, or activated NK cells; total or activated CD4^+^ T cells; total or activated CD8^+^ T cells; or PD-1^+^ T cells (Supplemental Figure 7, data not shown).

### Circulating levels of cytokines/chemokines associated with MDSC migration and recruitment.

**Figure 4:**
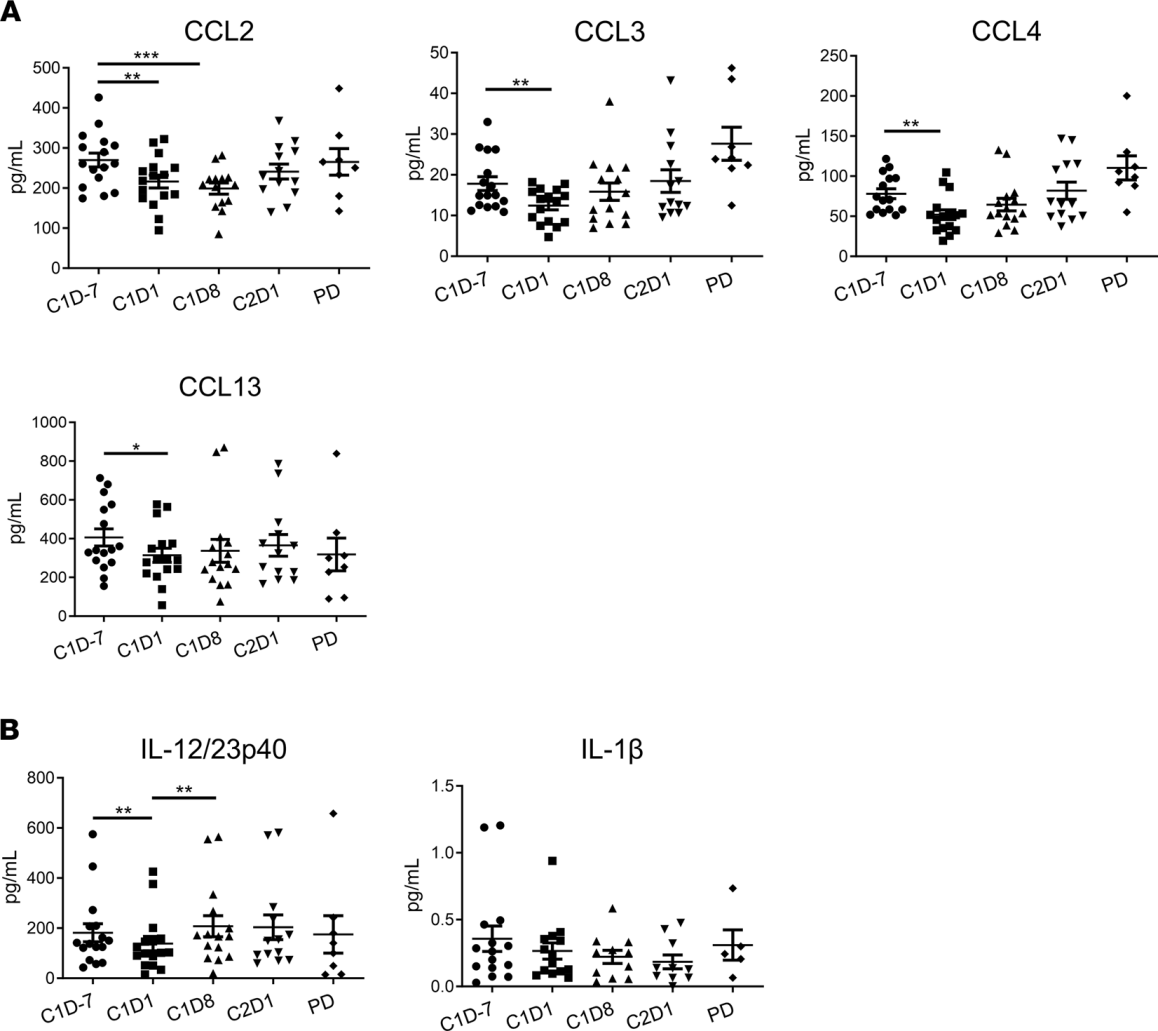
Circulating levels of chemokines and cytokines associated with MDSC migration and recruitment. Plasma levels of 20 cytokines and chemokines were measured at the indicated times and at progression of disease (PD) using a custom U-PLEX Human Cytokine Panel 20-plex Assay. The assay was performed in duplicate, and analyte levels were measured for all patients (*n* = 16) and displayed as mean ± SEM. (**A**) Levels of CCL2, CCL3, CCL4, and CCL13. (**B**) Levels of IL-12/23p40 and IL-1β. Data are analyzed by Student’s *t* test (paired), and *P* values are adjusted for multiple comparisons within each biomarker using Holm-Bonferroni method. *P < 0.05, **P < 0.01, ***P < 0.001.

Levels of 20 cytokines and chemokines were measured in plasma samples collected at C1D–7, C1D1, C1D8, C2D1, and the time of disease progression (Figure 4 and Supplemental Figure 8). Plasma levels of several factors associated with MDSC recruitment and accumulation were decreased in response to single-agent ibrutinib compared with baseline. Significant reductions were seen for CCL2 (270.0 ± 17.40 pg/mL to 216.3 ± 15.88 pg/mL, *P* = 0.005), CCL3 (17.83 ± 1.69 pg/mL to 12.45 ± 1.07 pg/mL, *P* = 0.001), CCL4 (78.04 ± 6.13 pg/mL to 51.96 ± 6.03 pg/mL, *P* = 0.002), and CCL13 (406.2 ± 43.94 pg/mL to 314.4 ± 36.36 pg/mL, *P* = 0.04; Figure 4A). Levels of the IL-12 and IL‑23 p40 subunit were also significantly decreased following ibrutinib treatment (181.8 ± 35.94 pg/mL to 138.4 28.41 pg/mL, *P* = 0.006; Figure 4B). Levels of IL-1β (0.36 ± 0.09 pg/mL to 0.2 ± 0.06 pg/mL, *P* = 0.58) were decreased with ibrutinib but not significantly (Figure 4B). Moreover, CCL2, CCL3, CCL4, IL-6, IL‑8, IL-1β, IL-12p70, TNF-α, and VEGF-A levels were significantly decreased at C2D1 in patients with clinical benefit (all *P* < 0.05) compared with patients with PD (Supplemental Figure 9).

### Differential gene expression profiles after treatment.

**Figure 5:**
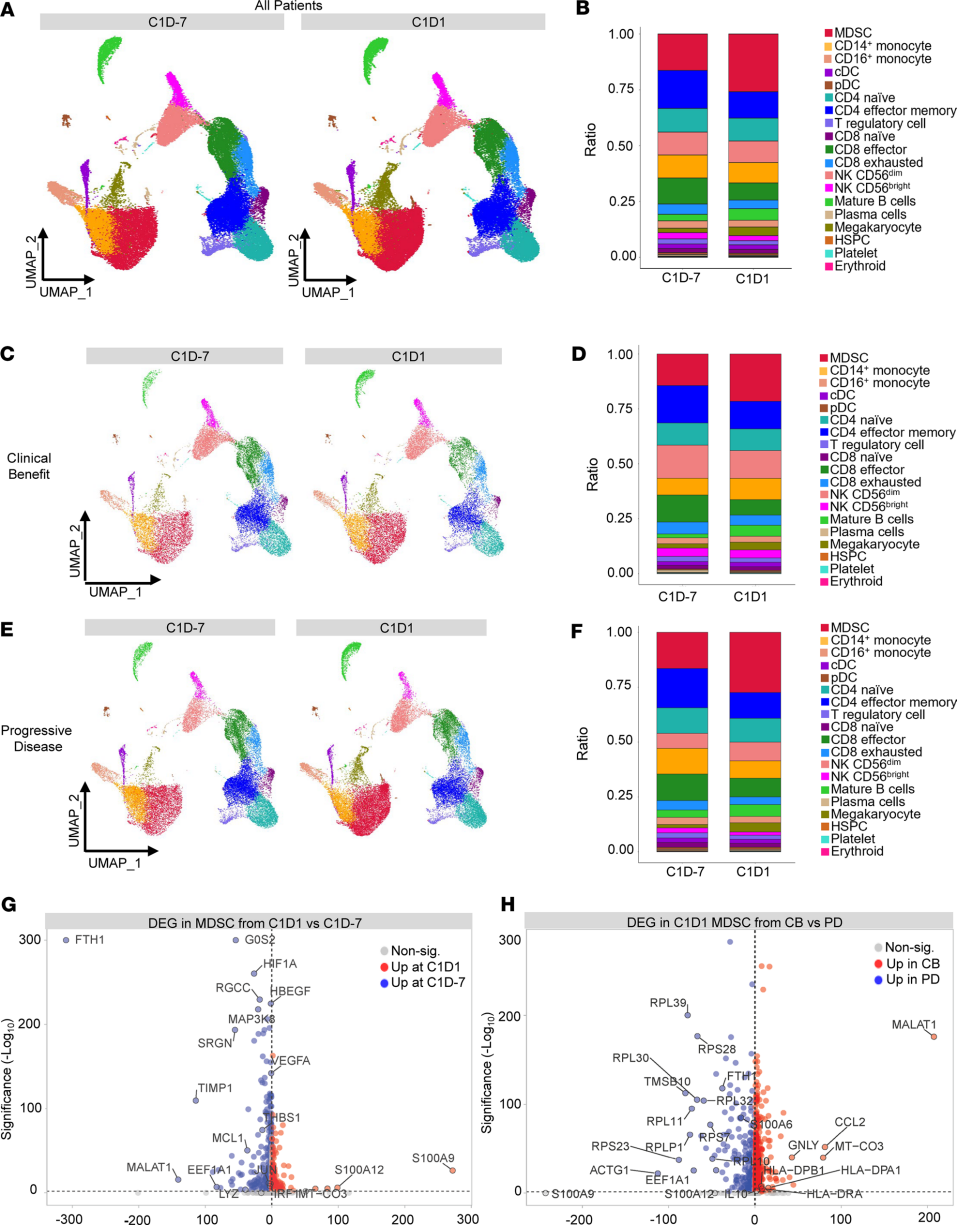
Single-cell RNA-Seq of patient immune cells after ibrutinib treatment and MDSC-specific gene changes. Peripheral blood mononuclear cell (PBMC) samples (2 paired samples from 14 patients plus individual samples from 2 patients, *n* = 30) at baseline (C1D–7) and following single-agent ibrutinib treatment (C1D1) were analyzed by scRNA-Seq. PBMC samples were aggregated and clustered into immune populations based on gene expression and visualized using UMAP. (**A**) UMAP showing clusters from all patients combined at C1D–7 (left) and C1D1 (right). (**B**) The ratio of each cell type present at each time point. (**C**) Combined UMAP from patients with clinical benefit (CB) (partial response or stable disease) who had 2 paired samples (*n* = 6) at C1D–7 (left) and C1D1 (right). (**D**) The ratio of each cell type present at each time point. (**E**) Combined UMAP plot from patients with 2 paired samples who had disease progression (PD, *n* = 8) at C1D–7 (left) and C1D1 (right). (**F**) The ratio of each cell type present at each time point. (**G**) Volcano plot of top differentially expressed genes in MDSC from all patients after ibrutinib treatment. Genes downregulated in MDSC at C1D1 relative to C1D–7 are represented in blue, and genes upregulated in MDSC at C1D1 relative to C1D–7 are represented in red. (**H**) Volcano plot of top differentially expressed genes in C1D1 MDSC from patients with CB versus patients with PD. Genes downregulated in MDSC from patients with CB relative to MDSC from patients with PD at C1D1 are represented in blue, and genes upregulated in MDSC from patients with CB relative to PD are represented in red (*x* axis = log_2_ fold change/*y* axis = –log_10_[adjusted *P* value]). cDC, conventional dendritic cell; pDC, plasmacytoid DC; HSPC, hematopoietic stem cells.

To investigate changes in immune cell gene expression after BTK inhibition, single-cell RNA-Seq (scRNA-Seq) was performed on patient PBMCs at baseline (C1D–7) compared with PBMCs obtained after 7 days of ibrutinib (C1D1) (*n* = 16 patients, *n* = 28 paired samples and 2 unpaired samples). Samples were aggregated into a Uniform Manifold Approximation and Projection (UMAP) for dimension reduction at C1D–7 and at C1D1 (Supplemental Figure 10, A and B, and Figure 5A). Expression levels of canonical cell markers were used to annotate immune cell clusters (Supplemental Figure 10C). Immune population percentages were calculated at C1D–7 and C1D1 (Figure 5B). As a group, patients exhibited an increase in MDSC and a decrease in CD4^+^ and CD8^+^ effector T cells. The exhausted CD8^+^ T cell subset decreased slightly after 7 days of BTK inhibition; however, this change was not significant (Figure 5B). Expression of several T cell exhaustion-related genes within the exhausted CD8^+^ T cell subcluster (*PDCD1*, *HAVCR2*, *LAG3*, *TIGIT*, *KLRG1*, *TOX*, *CTLA4*, *CD244*, and *TCF7*) were also largely unchanged at C1D1 (data not shown). However, individual patient– and tumor type–specific UMAPs suggested that patients did not respond to ibrutinib therapy uniformly (Supplemental Figures 11 and 12). Therefore, we chose to explore whether any consistent immunological changes occurred in patients who experienced similar clinical responses. The UMAP from patients with PD showed a distinct increase in MDSC levels, whereas patients with clinical benefit showed a smaller increase in MDSC levels at C1D1 (Figure 5, C–F). Patients with clinical benefit also had an increase in the CD14^+^ monocyte population at C1D1, while patients with PD had a decrease.

To better understand which immune cell population differed the most between patients with clinical benefit and patients with PD, we compared their overall gene expression profiles. MDSC had the most differentially expressed genes (DEG) between the 2 response groups with 1,891 genes differing between patients with clinical benefit and patients with PD (Supplemental Figure 13). MDSC analyzed collectively from all patients had 612 significantly DEGs after 7 days of ibrutinib therapy, with 171 genes being significantly upregulated and 441 genes being significantly downregulated. The top DEG are shown in the volcano plot in Figure 5G, and the top 15 up- and downregulated genes are listed in Supplemental Table 7. The top 5 upregulated genes at C1D1 were *S100A9*, *S100A12*, *MT-CO3*, *MT-CO1*, and *MT-CYB*, and the top 5 downregulated genes were *FTH1*, *MALAT1*, *TIMP1*, *B2M*, and *EEF1A1* (Supplemental Table 7). Several genes implicated in MDSC-suppressive functions (*TGFB1*, *HIF1A*, *VEGFA*, *THBS1*; ref. 22, 23), migration (*CXCL8*; ref. 24), survival (*MCL1*; ref. 25), and MDSC transcriptional regulation (*IRF1*; ref. 26) were also significantly downregulated following BTK inhibition (all *P* < 0.0002). C1D1 samples from patients with clinical benefit and patients with PD were then compared in order to investigate which MDSC genes differed the most in response to ibrutinib. C1D1 MDSC from patients with clinical benefit had significantly higher expression of *MALAT1*, which has been shown to be negatively correlated with the proportion of MDSC in lung cancer, several antigen presentation genes (*HLA-DPA1*, *HLA-DRA*, *HLA-DPB1*, *HLA-DRB1*); *GNLY*, an inducer of monocytic differentiation; and the chemokine *CCL2* than patients with PD (Figure 5H) (27–29). C1D1 MDSC from patients with PD had significantly higher upregulation of MDSC-associated genes (*S100A11*, *IL-10*, *FTH1*) and several ribosomal genes (*RPL39*, *RPS28*, *RPL30*, *RPL11*) than C1D1 MDSC from patients with clinical benefit (Figure 5H) (30–32). Subsequent pathway analysis performed using these DEG showed MDSC from patients with PD had significant enrichment in multiple pathways promoting increased cellular migration (all *P* < 0.02; Supplemental Figure 14).

CD8^+^ effector T cells also had a high number of DEG between patient response groups; thus, this population underwent further investigation as well (Supplemental Figure 13). Comparison of gene expression at C1D1 showed that CD8^+^ effector T cells from patients with clinical benefit had significantly higher expression of multiple ribosomal genes (*RPS27*, *RPS15A*, *RPL30*, *RPL29*) while patients with PD had significantly higher expression of *FTH1*, *GNLY*, and *ACTB* (Supplemental Figure 15A and Supplemental Table 8). Pathway analysis showed significant enrichment of metabolic processes, ribosome biogenesis, and RNA processing in patients with clinical benefit (all *P* < 0.0001) and significant enrichment of negative regulation of cell proliferation, endo- and phagocytosis, and FcγR signaling in patients with PD (all *P* < 0.05, Supplemental Figure 15B). The limited sample size in each response group emphasizes the exploratory nature of these analyses.

### MDSC ligand-receptor interactions are altered by ibrutinib therapy.

**Figure 6:**
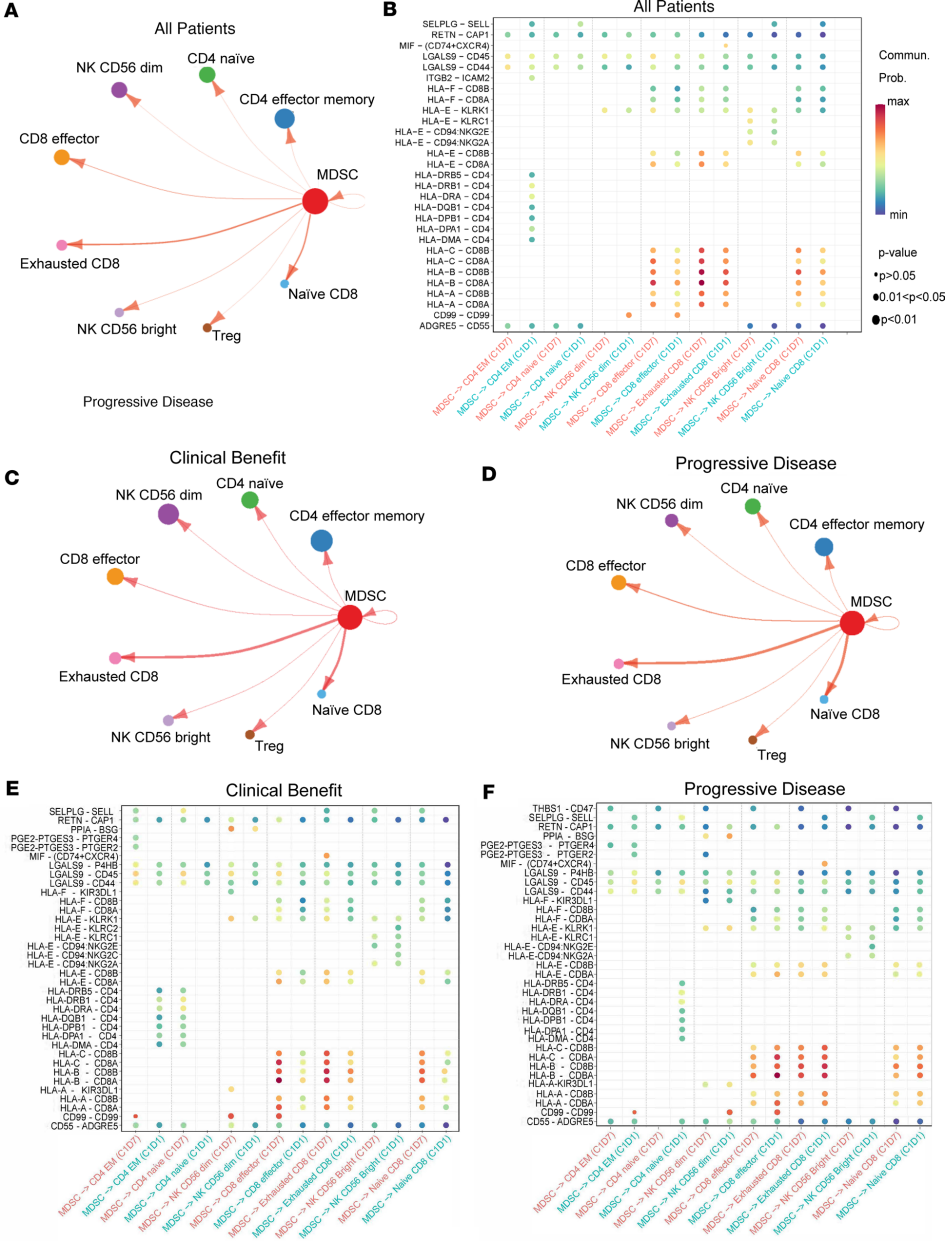
Analysis of changes in MDSC interactions and signaling networks after BTK inhibition. (**A**) Circle plot generated using CellChat analysis of single-cell RNA-Seq data depicting the inferred cell–cell communication networks between MDSC, T cells, and NK cells in all patients from C1D–7 and C1D1 combined (*n* = 16). Circle sizes are proportional to the number of cells in each group, and the weight of the arrows is proportional to the number of ligands-receptor pairs. (**B**) Significant ligand-receptor pairs between MDSC, T cells, and NK cells in all patients at C1D–7 and C1D1. Circle sizes are representative of *P* values, and the probability of communication is represented by color (minimal communication in blue, maximum communication in red). (**C**) Circle plot for patients with clinical benefit (partial response or stable disease, *n* = 6). (**D**) Circle plot for patients with progressive disease (*n* = 8). (**E**) Significant ligand-receptor pairs between MDSC, T cells, and NK cells in patients with clinical benefit at C1D–7 and C1D1. (**F**) Significant ligand-receptor pairs between MDSC, T cells, and NK cells in patients with progressive disease at C1D–7 and C1D1. Differential expression (DE) analyses were performed using the Wilcoxon rank-sum test. All *P* values were adjusted for multiple comparisons using a Bonferroni correction.

Signaling between MDSC and other immune cell populations is likely an important mechanism of MDSC-induced immunosuppression (3). CellChat (v1.4.; ref. 33) analysis was performed to investigate whether BTK inhibition affected the probability of specific MDSC-immune cell interactions using gene expression of known ligand-receptor pairs. These interactions are displayed in Figure 6A in a circle plot where the size of each circle is proportional to the number of cells in each population and the weight of the arrows is proportional to the number of known ligands-receptor pairs. The circle plot depicting the inferred cell-cell communication networks between MDSC, T cells, and NK cells in all patients revealed significant probabilities for MDSC interactions with CD4^+^ effector memory T cells, CD4^+^ naive T cells, CD56^dim^ NK cells, CD8^+^ effector T cells, exhausted CD8^+^ T cells, CD56^bright^ NK cells, Tregs, naive CD8^+^ T cells, and MDSC themselves. Specific ligand-receptor interactions before (C1D–7) and after 7 days of ibrutinib therapy (C1D1) were also evaluated (Figure 6B). The intercellular communication with the highest probability at baseline was between MDSC and exhausted CD8^+^ T cells, primarily driven by MHC class I from MDSC interacting with CD8A/B from exhausted CD8^+^ T cells. MDSC also had a high probability of communication with CD8^+^ effector and naive CD8^+^ T cells driven by this same MHC class I–CD8 ligand-receptor pair. Notably, the probability of these interactions decreased after 7 days of ibrutinib therapy. Contrastingly, the probability of an MHC class II–CD4 interaction between MDSC and CD4^+^ effector memory T cells was zero at baseline but significant by C1D1.

Based on the differential gene expression in MDSC from patients with clinical benefit and patients with PD, additional CellChat analyses were performed within these 2 response groups. While the circle plots of MDSC interactions with both T cells and NK cells were similar between the 2 response groups (Figure 6, C and D), there were notable differences in the probabilities of specific ligand-receptor interactions. The probabilities of MHC class I–CD8 interactions between MDSC and exhausted CD8^+^, CD8^+^ effector, and naive CD8^+^ T cells were decreased in patients with clinical benefit at C1D1 (Figure 6E) but increased in patients with PD (Figure 6F). Additionally, the probability of MHC class II–CD4 interactions between MDSC and CD4^+^ effector memory T cells was increased in patients with clinical benefit at C1D1 (Figure 6E), while patients with PD showed zero probability of such interactions before or after treatment (Figure 6F). There were also differences in the probabilities of MDSC macrophage migration inhibitory (MIF) signaling to exhausted CD8^+^ T cells and MDSC prostaglandin E2 (PGE2) signaling to CD4^+^ effector memory T cells between response groups (Figure 6, E and F). However, the small sample sizes of these patient response groups prevent definitive interpretation of these results without additional confirmation.

### Combination ibrutinib and nivolumab increases T cell proliferation.

**Figure 7:**
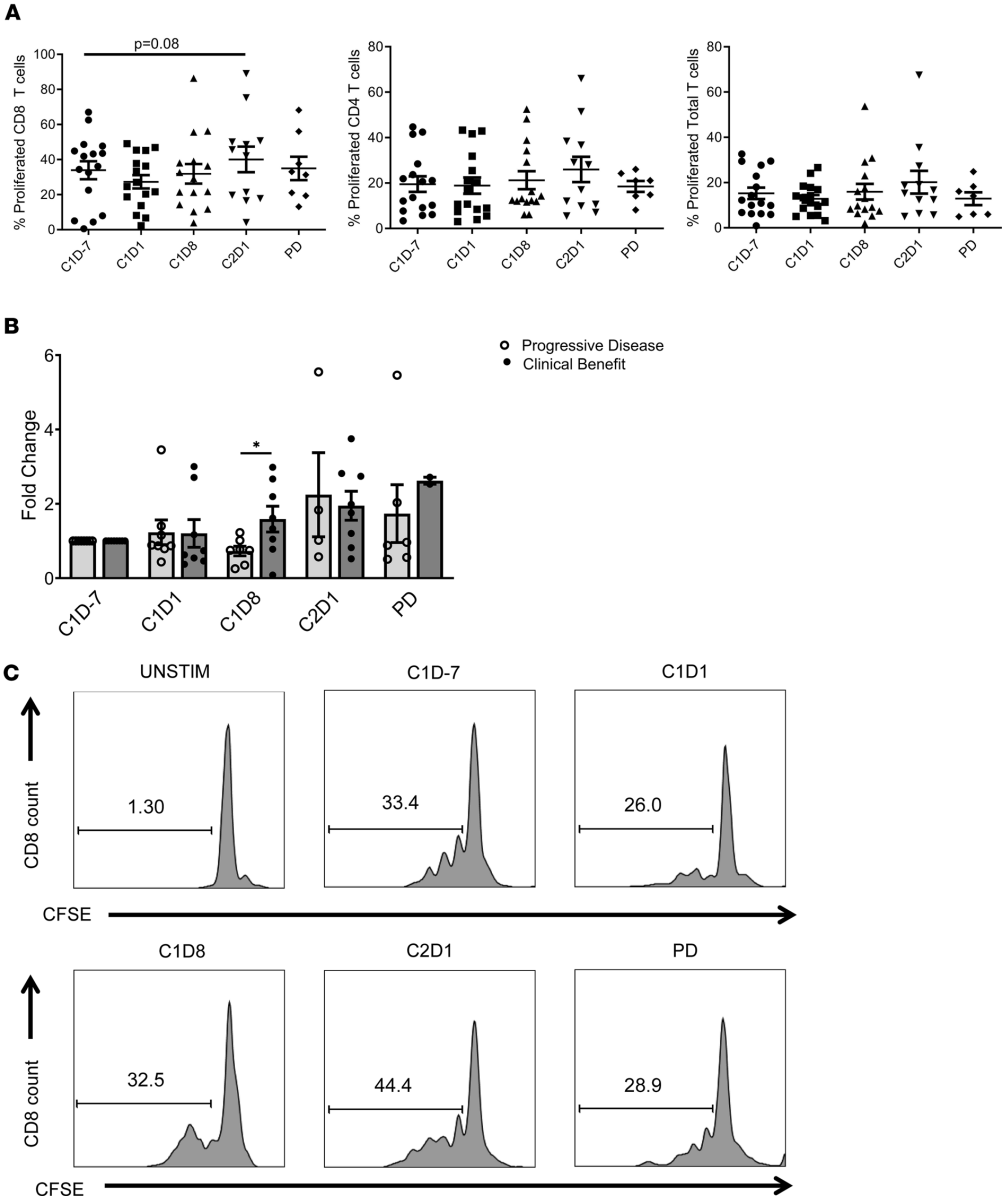
Ibrutinib in combination with nivolumab increases T cell proliferation. Patient PBMCs were activated with anti-CD3/CD28 beads and labeled with CFSE. After 3 days, cells were collected and stained with anti-CD8 and anti-CD4 antibodies; proliferation was assessed by flow cytometry. (**A**) Quantification of CD8^+^, CD4^+^, and total T cell proliferation from 16 patients. Data represent mean ± SEM. Student’s *t* test (paired) was used, and *P* values were adjusted for multiple comparisons using Holm-Bonferroni method. (**B**) T cell proliferation in patients that experienced clinical benefit (partial response and stable disease, *n* = 8) or progressive disease (*n* = 8) analyzed by Student’s *t* tests (unpaired). (**C**) Representative histograms of CD8^+^ T cell proliferation in 1 patient. *P < 0.05.

The effects of ibrutinib and nivolumab on T cell proliferation was measured throughout treatment; patient PBMC were CFSE-labeled, stimulated with anti-CD3/CD28 beads, and cultured for 72 hours. CD4^+^ and CD8^+^ T cell proliferation was measured by flow cytometry. In both populations, proliferation was not significantly changed from baseline to C1D1. However, CD8^+^ T cell proliferation increased following combination ibrutinib and nivolumab treatment at C2D1 compared with C1D–7 (33.93% ± 5.13% vs. 40.06% ± 7.31%, *P* = 0.08; Figure 7A). There was also a small increase in CD4^+^ T cell proliferation at C2D1 compared with C1D–7 (19.52% ± 3.42% vs. 25.98% ± 5.56%, *P* = 0.23; Figure 7A). Patients with clinical benefit exhibited a significant 1.6-fold increase in T cell proliferation from C1D–7 to C1D8 compared with patients with PD who exhibited a 0.7-fold decrease (*P* = 0.047). Notably, T cell proliferation was improved in both groups after the addition of nivolumab therapy (C2D1) compared with baseline (Figure 7B). Representative T cell proliferation histograms are presented in Figure 7C.

### TCR repertoire assessment.

**Figure 8:**
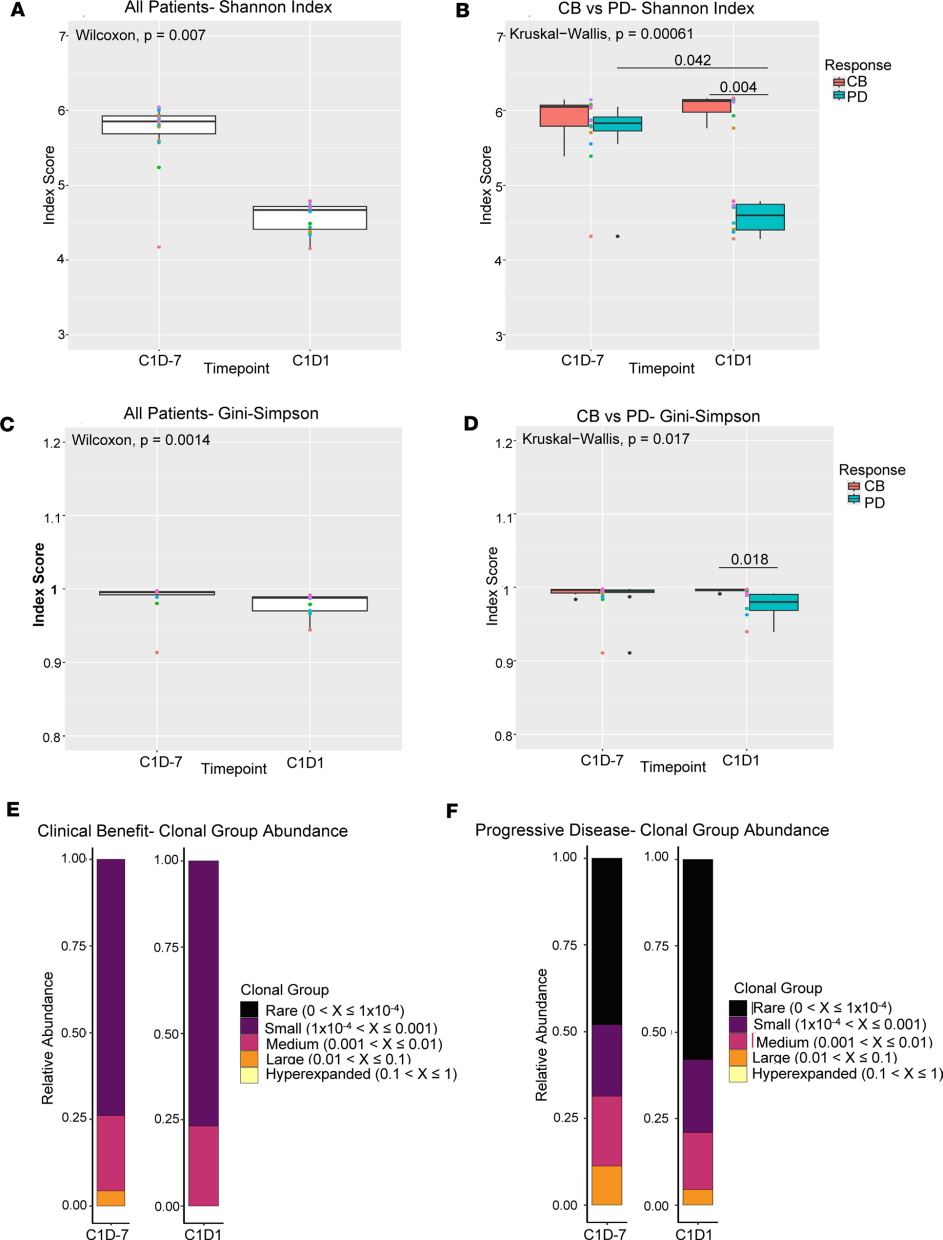
TCR repertoire diversity changes after ibrutinib treatment. The TCR-α and TCR-β CDR3 regions of 16 patients were amplified and sequenced using the 10X Genomics single cell immune profiling kit before (C1D–7) and after single-agent ibrutinib treatment (C1D1). (**A** and **B**) Shannon Diversity Index scores at C1D–7 and C1D1 in all patients (*n* = 16) (**A**) and patients with clinical benefit (CB, *n* = 6) or progressive disease (PD, *n* = 8) (**B**). (**C** and **D**) Gini-Simpson index scores in all patients (**C**) and patients with CB or PD (**D**). (**E** and **F**) Distribution of clonal type groups shown as relative abundance of rare, small, medium, large, and hyperexpanded clonal groups at C1D–7 and C1D1 in patients with CB (**E**) and patients with PD (**F**). The statistical significance of differences between response groups and/or time points was evaluated using the Wilcoxon rank-sum test for 2-group comparisons and the Kruskal-Wallis test for comparison of more than 2 groups. *P* values were adjusted for multiple testing using the Bonferroni procedure when multiple pairs were simultaneously evaluated. Boxes in the box-and-whisker plot represent the 25th and 75th percentiles, and the lines inside the boxes represent the median. Whiskers extend to the minimum and maximum values, and dots outside the whiskers represent outliers.

Patient TCR profiles have been studied in the context of cancer immunotherapy as a way to monitor T cell dynamics (34). Additionally, due to the predominant effect of ICIs on the T cell compartment, TCR-Seq is a promising candidate for predictive biomarkers of response to ICI therapy (35). Previous studies have correlated TCR repertoire diversity with responses to ICI therapy and shown increased TCR diversity following anti–PD-L1 and anti–PD-1 therapy in responders and patients with longer OS in non–small cell lung cancer and Merkel cell carcinoma (36, 37). As an exploratory objective, TCR repertoires were examined at baseline (C1D–7) and following 7 days of ibrutinib (C1D1). Shannon Diversity Index and Gini-Simpson index scores were calculated to assess TCR repertoire diversity. Analysis of Shannon Diversity Index scores before and after ibrutinib therapy in all patients showed a significant decrease following treatment (*P* = 0.007; Figure 8A). However, index score evaluation in the context of patient response showed that patients with clinical benefit had a nonsignificant increase in Shannon Diversity Index scores following ibrutinib therapy, while patients with PD had a significant decrease (*P* = 0.042; Figure 8B). This significant decrease in patients with PD may indicate a reduction of TCR repertoire diversity in response to 7 days of ibrutinib therapy (38). The Shannon Diversity Index score at C1D1 in patients with clinical benefit was also significantly higher than the C1D1 score in patients with PD (*P* = 0.004), suggesting that patients with clinical benefit had significantly higher TCR repertoire diversity at C1D1 than patients with PD. Similar results were seen using the Gini-Simpson index, with a significant decrease following ibrutinib therapy in all patients analyzed together (*P* = 0.0014; Figure 8C) and a significantly higher index score at C1D1 in patients with clinical benefit compared with patients with PD (*P* = 0.018; Figure 8D). Further analysis of the clonal group distributions within patient response groups highlighted the differences in clonal group abundances as well. The TCR repertoire of patients with clinical benefit was dominated by small clones (Figure 8E), while in patients with PD, the rare clonal group was most dominant (Figure 8F). Additionally, while both response groups had a reduction in the frequency of large clones following ibrutinib therapy, patients with PD had an expansion of the rare clonal group and patients with clinical benefit had an expansion of the small and medium clonal groups (Figure 8, E and F).

## Discussion

A pilot study of ibrutinib and nivolumab was conducted in patients with metastatic solid tumors. Ibrutinib in combination with nivolumab was well tolerated in this patient population. There was a diversity of tumor types, many of which do not have FDA-approved ICI treatment regimens, and 44% of patients had more than 1 prior line of therapy. Analysis of plasma following single-agent ibrutinib therapy revealed significantly decreased levels of chemokines associated with MDSC migration and recruitment (CCL2, CCL3, CCL4, CCL13). Mass cytometry demonstrated that MDSC levels decreased after single-agent ibrutinib and increased at the time of disease progression. The proportion of M-MDSC also significantly increased after 7 days of BTK inhibition, and the proportion of G-MDSC decreased. scRNA-Seq revealed ibrutinib-induced downregulation of several genes associated with MDSC-suppressive functions (*TIMP1*, *CXCL8*, *VEGFA*, *HIF1A*), a reduction in the probability of MDSC interactions with exhausted CD8^+^ T cells, and a decrease in average TCR repertoire diversity. The addition of nivolumab to ibrutinib therapy led to significant increases in circulating CD8^+^ T cells and NK cells, as well as an increase in CD8^+^ T cell proliferation. In an exploratory analysis according to patient response, partially responding patients were found to have the highest baseline levels of MDSC and the greatest overall reduction in MSDC levels following ibrutinib treatment. Overall, clinical benefit was observed in 8 (50%) patients, with 4 experiencing a PR. These results demonstrate the immunomodulatory effects of ibrutinib in patients with metastatic solid tumors and indicate a potential role of BTK inhibition in targeting MDSC.

Ibrutinib in combination with nivolumab was well tolerated. Grade 2 and grade 3 AEs were reported in 5 (31%) and 6 (38%) patients, respectively. Fatigue and anorexia represented the most common toxicities, and no dose reductions were required. No grade 4 or 5 events were reported. Ibrutinib-associated AEs included atrial fibrillation, thrombocytopenia, and maculopapular rash. Nivolumab-related symptoms included fatigue, rash, nausea, and diarrhea. The safety profile of the combination regimen was similar to that reported for the single agents (28, 32).

Efficacy was not the primary objective of this study; however, 4 of 16 patients achieved PR and 4 achieved SD. PR were seen in 3 of 4 patients with a tumor type that was FDA approved for nivolumab monotherapy, and benefit was observed in 5 patients bearing tumors that did not have approval for anti–PD-1 agents (1 PR and 4 SD). Another study of ibrutinib/nivolumab with cetuximab in patients with recurrent and/or metastatic head and neck squamous cell carcinoma (NCT03646461) is ongoing, and a study of ibrutinib with nivolumab for patients with previously treated kidney cancer (NCT02899078) has recently been completed (39). In the setting of metastatic renal cell cancer, it was found that administration of ibrutinib with nivolumab was safe. Antitumor activity was observed in a small subset of patients previously treated with anti–PD-1 therapy. An overall response rate of 10.7% was achieved; however, the target PFS rate was not reached, and no immune correlates were evaluated (19). Another study of ibrutinib/nivolumab in relapsed non-Hodgkin’s lymphoma and CLL (NCT02329847) found the combination to have an acceptable safety profile and clinical activity similar to single-agent ibrutinib. However, a promising clinical response was seen in a subset of patients with Richter’s transformation (40). Combination ibrutinib and nivolumab therapy also showed promising results in patients with refractory or relapsed central nervous system lymphoma (NCT03770416) with an 18-month PFS rate of 47% (41). Again, correlative immune-based assays were limited in these 2 studies. Collectively, these results have provided information on the safety and efficacy of this combination in patients with cancer, while the present study provides detailed information on the short-term effects of BTK inhibition. Additional work by Gunderson et al. demonstrates the immunomodulatory capacity of ibrutinib in myeloid cells and the ability of BTK inhibition to promote an increased CD8^+^ T cell immune response in pancreatic ductal adenocarcinoma (PDAC) (42). However, in a 424-patient phase III study from Tempero et al., ibrutinib in combination with nab-paclitaxel and gemcitabine chemotherapy in patients with PDAC resulted in no significant improvement in OS (43). This result may indicate a reduced potential for a response to ibrutinib therapy when combined with chemotherapy, given the immune-suppressive effects of cytotoxic agents.

There is literature describing the inhibitory effects of MDSC in cancer, highlighting this population as an attractive therapeutic target (11). In the present study, average MDSC levels measured by mass cytometry decreased after 7 days of ibrutinib therapy and increased at the time of disease progression. The proportion of the M-MSDC subset of MDSC significantly increased after single-agent ibrutinib while the proportion of the G-MDSC subset decreased. M-MDSC represented the predominant subset of circulating MDSC across all time points, consistent with literature reports on circulating MDSC subsets in melanoma and prostate cancers (44). scRNA-Seq indicates that MDSC gene expression was also affected by ibrutinib. There was a total of 612 DEGs in the MDSC cluster after 7 days of BTK inhibition. Genes implicated in MDSC-suppressive functions (*TIMP*, *TGFB1*, *HIF1A*, *VEGFA*, *THBS1*; refs. 22, 23), migration (*CXCL8*; ref. 24), survival (*MCL1*; ref. 25), and transcriptional regulation (*IRF1*; ref. 26) were significantly downregulated following BTK inhibition. The 2 most significantly upregulated genes in MDSC after ibrutinib therapy were *S100A9* and *S100A12*. These 2 genes are known to be expressed in MDSC and are implicated in the suppressive functions of M-MDSC via their ability to contribute to MDSC trafficking, activation, and T cell suppression (45, 46). Notably, levels of *S100A9* were 243-fold higher in patients with PD after ibrutinib therapy compared with patients with clinical benefit, and *S100A12* levels were 47-fold higher. This suggests that the MDSC response to BTK inhibition may correlate with patient responses to therapy. Furthermore, analysis of gene expression differences between PD and patients with clinical benefit highlight MDSC as the immune cell population with the most differential gene expression between response groups.

Based on these findings, an exploratory analysis was performed to determine if additional gene expression differences could be detected in MDSC from patients with differing clinical responses. Analysis of MDSC in patients with clinical benefit compared with those with PD demonstrated disparate gene changes following 7 days of ibrutinib. Ibrutinib-treated MDSC from patients with clinical benefit had significantly higher expression of several MHC class II antigen presentation genes (*HLA-DPA1*, *HLA-DRA*, *HLA-DPB1*, *HLA-DRB1*) than MDSC from patients with PD. MDSC can differentiate into antigen-presenting cells through an increase in MHC class II expression (2), suggesting MDSC from patients with clinical benefit may have differentiated. Additionally, ibrutinib-treated MDSC from patients with PD had significantly higher expression of several ribosomal genes (*RPL39*, *RPS28*, *RPL30*, *RPL11*) compared with MDSC from patients with clinical benefit. Downregulation of ribosomal RNA transcription has been shown to trigger cell differentiation in multiple settings, including in a human acute myeloid leukemia cell line and in mouse hematopoietic stem cells (47). Thus, the significantly higher expression of ribosomal genes in MDSC from patients with PD, in conjunction with the significantly lower expression of MHC class II genes, may indicate a difference in MDSC differentiation states between response groups (48).

Ibrutinib-treated CD8^+^ effector T cells from patients with clinical benefit also had significantly higher expression of ribosomal genes than those from patients with PD. This was the opposite of what was seen in MDSC. However, unlike in MDSC, translational activity of ribosomal proteins has been shown to be upregulated in CD8^+^ T cells during activation and clonal expansion (49). An increase in metabolism also occurs in CD8^+^ T cells upon antigenic stimulation of the TCR and promotes proliferation and effector functions (50). Thus, the increase in ribosomal genes and enrichment of pathways associated with ribosome biogenesis and metabolic processes in CD8^+^ effector T cells from patients with clinical benefit may indicate an increased level of activation (51).

CellChat analysis of differential gene expression revealed distinct changes in MDSC-immune cell interactions following BTK inhibition as well. The interaction with the highest probability at baseline was between MDSC and CD8^+^ T cells (exhausted, effector, and naive) primarily driven by MHC class I from MDSC interacting with CD8A/B from CD8^+^ T cells. The probabilities of these interactions were reduced following 7 days of ibrutinib therapy. MDSC MHC class I antigen presentation to CD8^+^ T cells has been shown to be important for their inhibitory functions (52); thus, the decrease in interaction probability may indicate a change in MDSC-suppressive ability following BTK inhibition. Using PD-1 expression as 1 marker of T cell exhaustion, it was shown by mass cytometry that single-agent ibrutinib had no significant effect on this parameter. Evaluation of the exhausted CD8^+^ T cell subcluster in the scRNA-Seq data set confirmed this finding. The reduction in MDSC interactions with exhausted CD8^+^ T cells is therefore likely an MDSC-centered effect and not due to a reduction in the exhausted CD8^+^ T cell subset.

In line with the gene expression differences in MDSC from patients with clinical benefit and patients with PD, there were differences in the probabilities of specific MDSC ligand-receptor interactions as well. The probability of MHC class I–CD8 interactions between MDSC and exhausted, effector, and naive CD8^+^ T cells was decreased in patients with clinical benefit but increased in patients with PD. This may indicate a more T cell–suppressive phenotype in MDSC from patients with PD. The probability of MHC class II–CD4 interactions between MDSC and CD4^+^ effector memory T cells was also increased in patients with clinical benefit, while patients with PD showed zero probability of such interactions at baseline or after treatment**.** This correlates with the scRNA-Seq finding that MDSC from patients with clinical benefit had higher expression of MHC class II antigen presentation genes than MDSC from patients with PD. This may indicate an increased capability for MDSC antigen presentation to CD4^+^ T cells in patients with clinical benefit; this increase has also been found in ex vivo MDSC treated with ibrutinib and is correlated with MDSC differentiation into mature DCs (53). Collectively, these findings suggest that there may be differences in the interactions of MDSC after BTK inhibition.

TCR-Seq demonstrated that ibrutinib also led to changes in the peripheral TCR repertoire, with a significant decrease in TCR repertoire diversity following 7 days of treatment. Investigation into differences between patient response groups, however, revealed a significantly lower TCR repertoire diversity in patients with PD than in patients with clinical benefit following BTK inhibition. Several studies have shown that circulating TCR repertoire diversity is reduced during carcinogenesis and metastasis (38, 54), while ibrutinib and PD-L1 blockade have both been shown to increase TCR diversity (36, 55). Furthermore, patients with increased TCR diversity after PD-L1 blockade were found to have significantly longer OS than those who had decreased diversity (36). This aligns with the current study, in which patients who developed disease progression had a significant reduction in TCR repertoire diversity while patients with sustained responses had no significant changes in diversity. A study by Yin et al. of ibrutinib monotherapy or ibrutinib plus rituximab found that TCR repertoire diversity significantly increased after ibrutinib therapy, though this analysis was performed after 1 year of ibrutinib treatment compared with just 7 days of treatment in the current study (56). The effect of MDSC inhibition on the T cell compartment and TCR repertoire is under active investigation by our group.

The primary limitation of this study was the relatively small number of patients and the heterogeneity of the tumor types. The majority of patients had received prior therapy, which could have led to diminished immune responses. Additionally, 12 of the 16 total patients had a tumor type for which nivolumab was not yet approved. However, the finding of significant changes in MDSC gene expression and immune-cell interactions in this diverse patient group highlights the effect of BTK inhibition on immune function. This also suggests that there may be a common immune-based response to ibrutinib therapy among multiple solid tumor types. This study may therefore be used as a pilot to encourage and inform the conduct and analysis of future ibrutinib studies in solid tumors.

In conclusion, ibrutinib and nivolumab was well tolerated and showed an acceptable safety profile in patients with metastatic solid tumors. The preliminary clinical activity seen in this study was accompanied by alterations in MDSC levels and MDSC gene expression, as well as modulation of the T cell compartment. This study provides biological evidence to support the continued development of strategies to target MDSC in combination with ICIs.

## Methods

### Sex as a biological variable.

Males (56%) and females (44%) participated in the clinical trial; sex was not considered as a biological variable.

### Study design.

This single-arm, pilot study (NCT03525925) was conducted at The Ohio State University Comprehensive Cancer Center beginning in August 2018. Eligible participants were treated with ibrutinib at the recommended phase 2 dose of 420 mg given orally daily for 15 days. Nivolumab was given at a standard dose of 240 mg i.v. over 30 minutes on days 1 and 15 of the 28-day cycle. Treatment was given on an outpatient basis. Ibrutinib started 7 days prior to cycle 1 of nivolumab and was given with nivolumab until day 8 of cycle 1 for a total of 15 days. After cycle 1, the dose of nivolumab could be changed to 480 mg i.v. once every 28 days, as desired by the treating physician or patient. Peripheral blood was collected prior to initiation of ibrutinib and prior to drug administration on days 1 and 8 of cycle 1, day 1 of cycle 2, and at the time of disease progression (Supplemental Table 1).

### Study objectives.

The primary objective was to assess the tolerability of ibrutinib and nivolumab in patients with advanced solid tumors. The secondary objective was to evaluate the effect of ibrutinib on circulating levels of MDSC. Exploratory objectives were to evaluate the effect of ibrutinib and nivolumab on MDSC function and T cell activity as well as to preliminarily evaluate the effect of the regimen on PFS.

### Patient population.

Eligible patients were adults (≥ 18 years old) with biopsy-proven metastatic solid tumors who were candidates to receive nivolumab per the treating physician. Patients could have had any number of prior therapies. Patients with measurable and nonmeasurable disease were permitted. Other eligibility criteria were ECOG performance score of 0–2 (57); life expectancy ≥ 12 weeks; and adequate BM, hepatic, and renal function. Exclusion criteria included history of nivolumab or ibrutinib use, autoimmune disease (except for treated Hashimoto’s thyroiditis), untreated/uncontrolled CNS metastases, interstitial pulmonary disease, prior systemic chemotherapy within 3 weeks, or systemic steroids within 5 days prior to study initiation.

### Sample collection.

In total, 40 mL of peripheral blood was collected at each time point (Supplemental Table 1). Peripheral blood mononuclear cells (PBMCs) were isolated from peripheral blood via Ficoll density gradient centrifugation (445*g*), as described (58). Briefly, samples were layered on Ficoll density gradient medium and centrifuged for 25 minutes at 445*g* with the brake off. Enriched populations were removed from the density gradient medium. PBMCs were cryopreserved for future analyses. Plasma samples were stored at −80°C until analysis.

### Clinical assessments.

Patients underwent screening laboratories within 2 weeks prior to initiating therapy. Baseline disease assessment was required within 4 weeks of study initiation. Routine laboratory tests (complete blood count with differential, complete metabolic panel, thyroid function, and tumor markers) were performed at C1D–7 and day 1 of each cycle as per standard of care. Toxicity assessments were performed on C1D–7, C1D1, C1D15, and C2D1. Common Toxicity Criteria for Adverse Events (CTCAE) version 4.0 was used to grade toxicities. Response/progression were evaluated using the revised Response Evaluation Criteria in Solid Tumors (RECIST) guideline (version 1.1). Patients were imaged every 3–4 weeks.

### Mass cytometry.

PBMCs were analyzed via mass cytometry (CyTOF) as previously described (59) using a 37-marker Maxpar Direct Immune Profiling Assay (Fluidigm) with additional custom-conjugated antibodies (Supplemental Table 5). Briefly, PBMCs were resuspended in cell staining buffer (CSB, Fluidigm) and Fc-receptor blocked with Human TruStain FcX (BioLegend). PBMCs were then stained and fixed in 1.6% formaldehyde solution. Following fixation, PBMCs were resuspended in Cell-ID Intercalator-Ir (Fluidigm) and incubated overnight at 4°C. Fixed PBMCs were washed twice with CSB and Maxpar Cell Acquisition Solution (CAS). Cells were resuspended in CAS containing 0.1× EQ Four Element Calibration beads (Fluidigm). Sample acquisition was performed on a Helios system utilizing CyTOF Software version 6.7.1016 using the Maxpar Direct Immune Profiling Assay template. At least 500,000 events were acquired per file. Data were normalized using CyTOF Software v.6.7.1016. Normalized FCS files were evaluated using Cytobank. PBMCs were gated in Cytobank to establish immune populations summarized in Supplemental Table 6. Representative gating strategies for CD45^+^ live cells and MDSC are presented in Supplemental Figure 3.

### Measurement of plasma cytokines and chemokines.

For quantitative detection of plasma cytokines, an unbiased pairwise screening of 20 analytes (IFN-γ, IL-1β, IL-2, IL-4, IL-6, IL-8, IL-10, IL-12p70, IL-13, TNF-α, GM- CSF, IL-5, IL-12/23p40, IL-17A, VEGF-A, CCL4, IP-10, CCL3, CCL2, and CCL13) was performed on U-PLEX plates using an electro-chemiluminescence method and read on the Meso QuickPlex SQ 120 (Meso Scale Discovery). All samples were run in batches, assayed in duplicate, and quantitated using a standard curve.

### scRNA-Seq and TCR-Seq read alignments and quality control.

PBMC from patients at C1D–7 and C1D1 were thawed, washed, and counted. Viability was 80%–92%. Single cells were isolated using the 10X Chromium Next GEM 5′ gene expression kit, targeting recovery of 4,000 cells/sample. Gene expression and TCR libraries were constructed using 10X Genomics Chromium Next GEM Single Cell Immune Profiling, v11.1 Chemistry, and sequenced on an Illumina NovaSeq according to manufacturer instructions (10X Genomics). Single-cell reads were aligned to GRCh38 (GENCODE v32/Ensembl 98) human reference using Cell Ranger (version 5.0.0, 10X Genomics). Raw expression data from 30 samples contained 114,268 cells total. In total, 13,088 outlier and low-quality cells with < 300 genes, 500 UMIs detected, or > 20% mitochondria percentage were excluded from analysis (60). The C1D–7 sample for patient 8 and C1D1 sample for patient 9 failed quality control on the initial library step of the RNA-Seq protocol and, thus, were excluded from further analysis.

### Cell clustering, doublet calling, and annotation.

Gene expression counts were normalized, log-transformed, and scaled by SCTransform in R software package Seurat (4.0.0) (61). Principal components were calculated from the top 2,000 variable genes, and the top 100 principal components were selected to generate UMAP (62). Clusters of similar cells were detected using the Louvain algorithm, and UMAP coordinates were used to construct a shared nearest neighbor graph by the FindNeighbors function at 0.6 resolution (63).

To identify cell type signatures, differential expression (DE) analysis between clusters was performed using MAST statistical framework implemented in the FindMarkers function (64) and adjusted by cellular detection rate. Wilcoxon rank-sum tests were used for DE analyses, and *P* values were adjusted for multiple comparisons using the Bonferroni correction. Genes with Bonferroni-corrected *P* < 0.05 were considered. Doublets were identified as cells with substantial and consistent expression profiles from 2 or more tissues and/or cell types. Single cell type and cluster annotation was performed using the SingleR (65) package and PanglaoDB (66), respectively.

### DE analysis and pathway enrichment analysis of scRNA-Seq data.

DE analysis between time points (C1D–7 and C1D1) and patient response groups (clinical benefit [*n* = 6] and PD [*n* = 8]) was performed using MAST statistical framework (2) implemented in the FindMarkers function (64) and adjusted by cellular detection rate. Wilcoxon rank-sum tests were used for DE analyses, and *P* values were adjusted for multiple comparisons using the Bonferroni correction. Genes with Bonferroni-corrected *P* < 0.05 were considered. Visualization of gene sets was conducted using ggpubr (0.4.0) and ggplot2 (3.3.5) R packages (67),

DEG between clinical benefit (*n* = 6) and PD (*n* = 8) patient C1D1 MDSC and CD8^+^ effector T cells were obtained using DE analysis in Seurat (4.0.0) using the MAST method (2) with UMI as a latent variable. DEG were analyzed by GO enrichment analysis using the fgsea function to identify canonical pathways overrepresented or underrepresented by the identified DEG. Enrichment is displayed as a normalized enrichment score (NES). Overrepresentation of a pathway was denoted by a positive NES and underrepresentation of a pathway was denoted by a negative NES. Statistical analyses were performed using the fgseaMultilevel function in the fgsea (1.16.0) R package. Pathways with a Bonferroni-corrected *P* < 0.05 were considered significantly enriched.

### Cell-cell interaction analysis.

To analyze cell-cell interactions between MDSC and other immune cells of interest, CellChat (v2) was used (68). MDSC interactions with CD4^+^ effector memory T cells, CD4^+^ naive T cells, CD56^dim^ NK cells, CD8^+^ effector T cells, exhausted CD8^+^ T cells, CD56^bright^ NK cells, Tregs, naive CD8^+^ T cells, and MDSC themselves were evaluated. Potential ligand-receptor interactions were derived based on the expression of a receptor by one cell subpopulation and ligand expression by another. DE analyses were performed using the Wilcoxon rank-sum test. All *P* values were then adjusted for multiple comparisons using a Bonferroni correction.

### T cell proliferation assay.

PBMCs were labeled with carboxyfluorescein succinimidyl ester (CFSE, Thermo Fisher Scientific, C34554) in 0.1% BSA for 20 minutes at 34°C as described (58). Labeled PBMCs were then washed with RPMI medium and plated at 2 × 10^5^ cells/well in triplicate. T cells within PBMCs were activated with anti‑CD3/CD28 beads (Thermo Fisher Scientific, 11161D), and PBMCs without bead stimulation were controls. After 72 hours, proliferation was assessed by flow cytometry. APC anti-CD4 (BioLegend, 357407) and PE-Cy7 anti-CD8 (BioLegend, 344711) antibodies were used to identify T cell subsets.

### TCR analysis.

VDJ analysis was implemented using the scRepertoire package (version 2.0.0) (69). Clonotypes were defined based on the genes comprising the TCR/Ig and the nucleotide sequence of the CDR3 region. Shannon Diversity Index scores (70) and Gini-Simpson index scores (71) were calculated using the ClonalDiversity function (72) for different patient response groups (clinical benefit and PD) and time points (C1D–7 and C1D1). The statistical significance of differences between response groups and/or time points was evaluated using the Wilcoxon rank-sum test for 2-group comparisons and the Kruskal-Wallis test for comparison of more than 2 groups. *P* values were adjusted for multiple testing using the Bonferroni procedure when multiple pairs were simultaneously evaluated. The relative abundance of clonal types was calculated using clonalHomeostasis function (69) and compared between response groups and time points. Clonotype groups were defined based on the frequencies (X) of detectable reads: rare (0 < X ≤ 1 × 10^–4^), small (1 × 10^–4^ < X ≤ 0.001), medium (0.001 < X ≤ 0.01), large (0.01 < X ≤ 0.1) and hyperexpanded (0.1 < X ≤ 1).

### Statistics.

Unless otherwise indicated, data are presented as mean ± SEM), and 2-tailed Student’s *t* tests or 1-way ANOVA were used to assess statistical significance. The Holm-Bonferroni method was used to adjust the multiple comparisons over time within each biomarker. *P* < 0.05 was considered statistically significant. Required patient sample size was calculated to be > 13 to provide at least 90% power to detect a mean of differences in MDSC levels of 3.3% with an α of 0.05 based on a 2-sided paired *t* test, assuming a 3.3% SD of the difference. Patient characteristics were summarized using descriptive statistics (count and percentage for categorical variables; mean, SD, median, and range). AEs were summarized by grade per the CTCAE, version 4.0 criteria. Progression-free survival (PFS) was measured as the time from treatment initiation to clinical or radiologic disease progression or death from any cause. Post hoc exploratory analysis of OS was also conducted. OS was defined as time from treatment initiation to death from any cause. Patients who were still alive were censored on March 1, 2021. PFS and OS were analyzed using the Kaplan-Meier method. For scRNA-Seq and TCR-Seq, the 2-tailed paired Wilcoxon rank-sum test was used to assess changes within patients before and after treatment. The 2-tailed unpaired Wilcoxon test was used to assess changes between patients. The Kruskal-Wallis test was used for comparison of more than 2 groups. *P* values were adjusted for multiple testing using the Bonferroni procedure when multiple pairs were simultaneously evaluated. Each patient was treated as 1 pseudo-bulk data point by calculating the average value for each time point. All statistical analyses were performed with the software R.

### Study approval.

This clinical trial is registered at https://register.clinicaltrials.gov (ClinicalTrials.gov, NCT03525925). The study was conducted at The Ohio State University Comprehensive Cancer Center under an IRB-approved protocol (IRB protocol no. 2018C0070). All patients were required to read and sign an IRB‑approved informed consent prior to screening and study-related procedures.

### Data availability.

Values for all data points in graphs are reported in the Supporting Data Values file. Raw sequencing data are available in GEO (accession no. GSE178882). Scripts used for analysis and generating scRNA-Seq figures are available at https://github.com/jianying609/Ibrutinib (commitID: 754b4973ff59c9dba651107b9e18862f56477b12). Scripts used for analysis and generating TCR-Seq figures are available at https://github.com/iamkj03/tcrseq_mdsc_analysis (commitID: 2068c2bea61295dcc1609891de185badf64e1f34).

## Author contributions

RW, KK, CV, BK, MS, WEC, BB, DQ, ES, KB, JH, RR, HAS, PM, and AMN assisted in the initiation, design, and execution of the study. BB, ES, GL, HS, LG, LS, MCD, JS, BWB, DQ, GB, and SHS conducted experiments and acquired data for this study. YH, OE, ES, WEC, HS, BB, BWB, OE, JL, KJJ, DC, and GX contributed to data analysis, and ES, BB, YH, XP, and LW assisted with statistical analysis. ES, BB, WEC, RW, and LW wrote and edited the manuscript.

## Supplementary Material

Supplemental data

ICMJE disclosure forms

Supporting data values

## Version 1 11/08/2024

Electronic publication
