# Supplementary material for: Inhibition of Bruton’s tyrosine kinase with PD-1 blockade modulates T cell activation in solid tumors
Source: JCI Insight. 2024 Nov 8;9(21):e169927. doi: 10.1172/jci.insight.169927 (PMC11601564; doi:10.1172/jci.insight.169927)
Supplement: Supplemental data [file jciinsight-9-169927-s045.pdf]

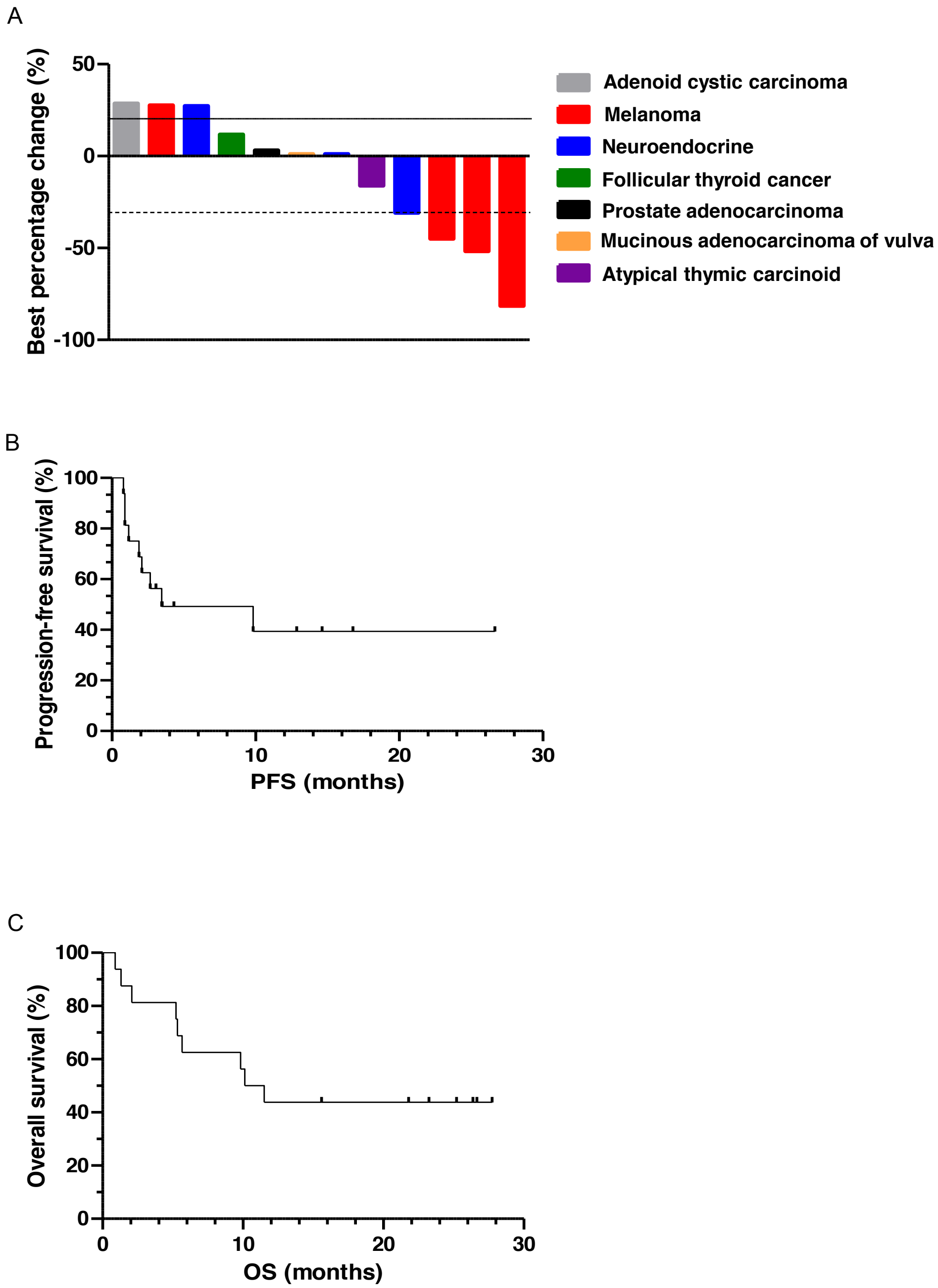

Supplemental Figure 1. Exploratory analysis of clinical efficacy. (A) Waterfall plot of best percent change from baseline for target lesions by RECIST v1.1 by tumor type (n=12 patients). Only patients with both baseline and post-baseline tumor assessments were included in this analysis. Kaplan-Meier curves for **(B)** progression-free survival (PFS) and **(C)** overall survival (OS).

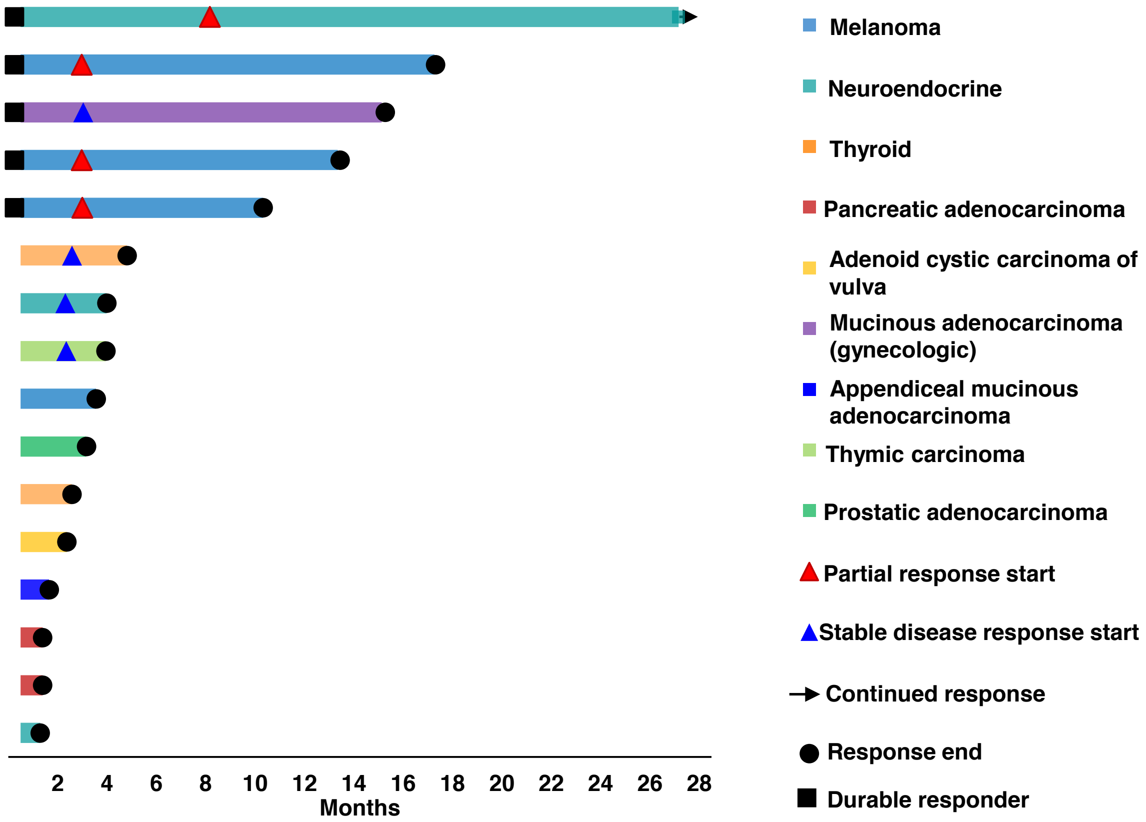

Supplemental Figure 2. Swimmer plot of tumor types and best overall responses. The onset and duration of partial response, stable disease, response end, the continuation of response and durable responder are indicated with specific symbols. A durable response is defined as any patient with a response of > 6 months. Patients are colored by tumor type.

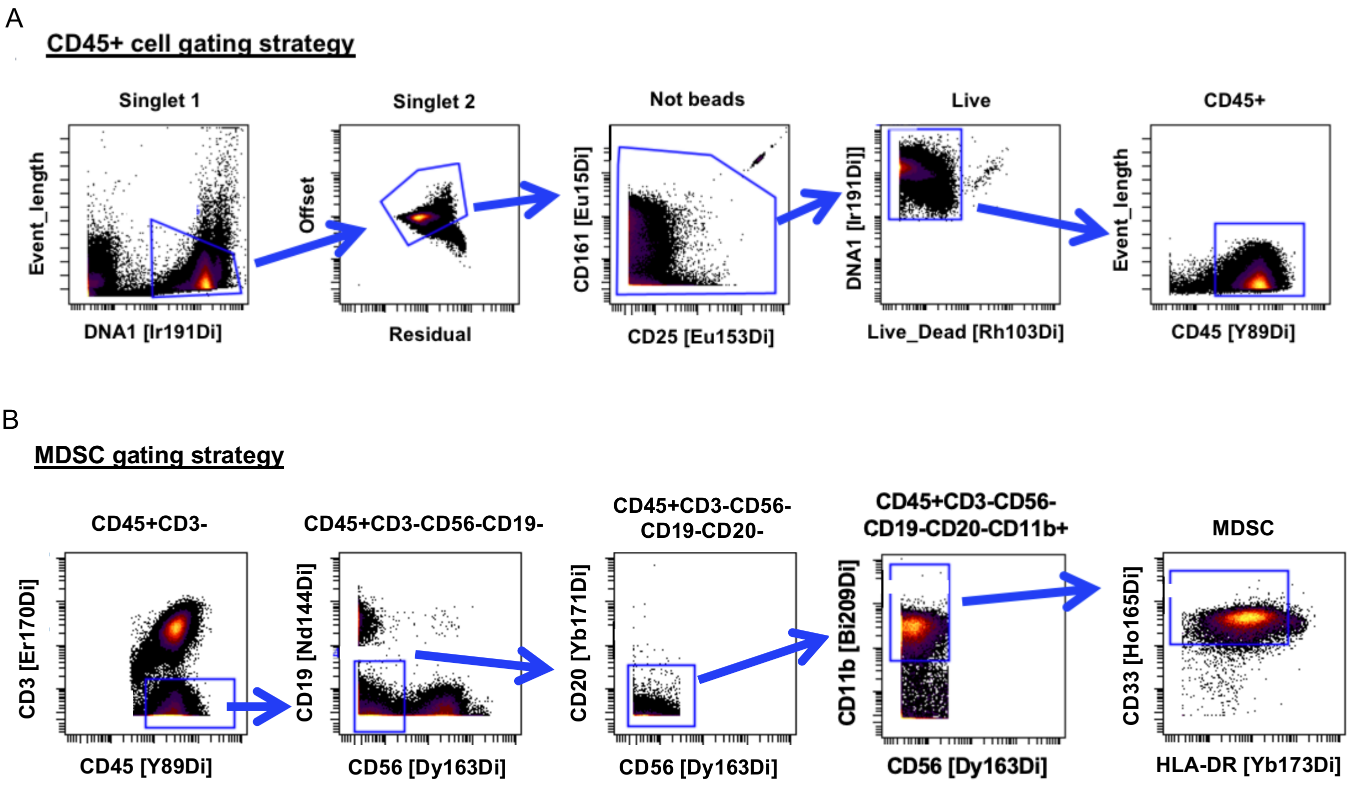

Supplemental Figure 3. MDSC gating strategy. (A) Mass cytometry gating strategy for isolation of live CD45^{+} cells from PBMC and (B) MDSC gating strategy from CD45^{+} cells.

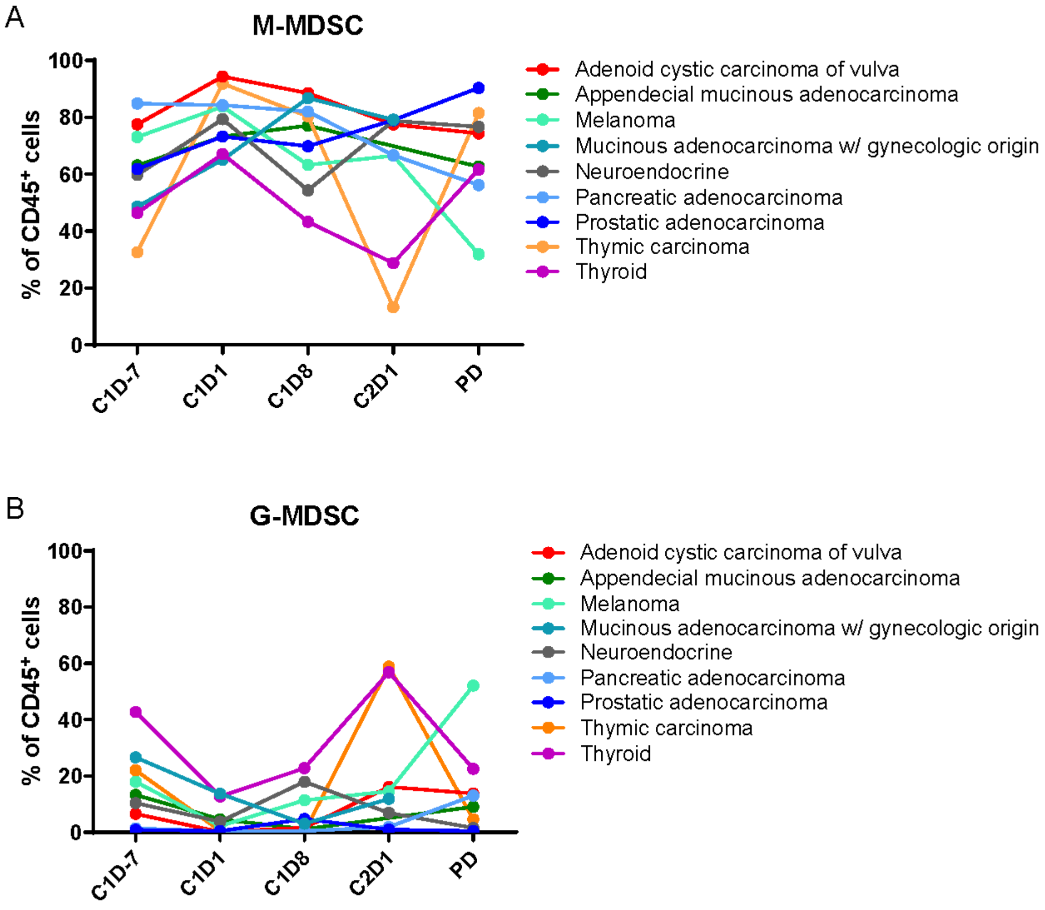

Supplemental Figure 4. MDSC subset levels according to tumor type. Subsets of MDSC, monocytic (M)-MDSC and granulocytic (G)-MDSC represented by tumor type.

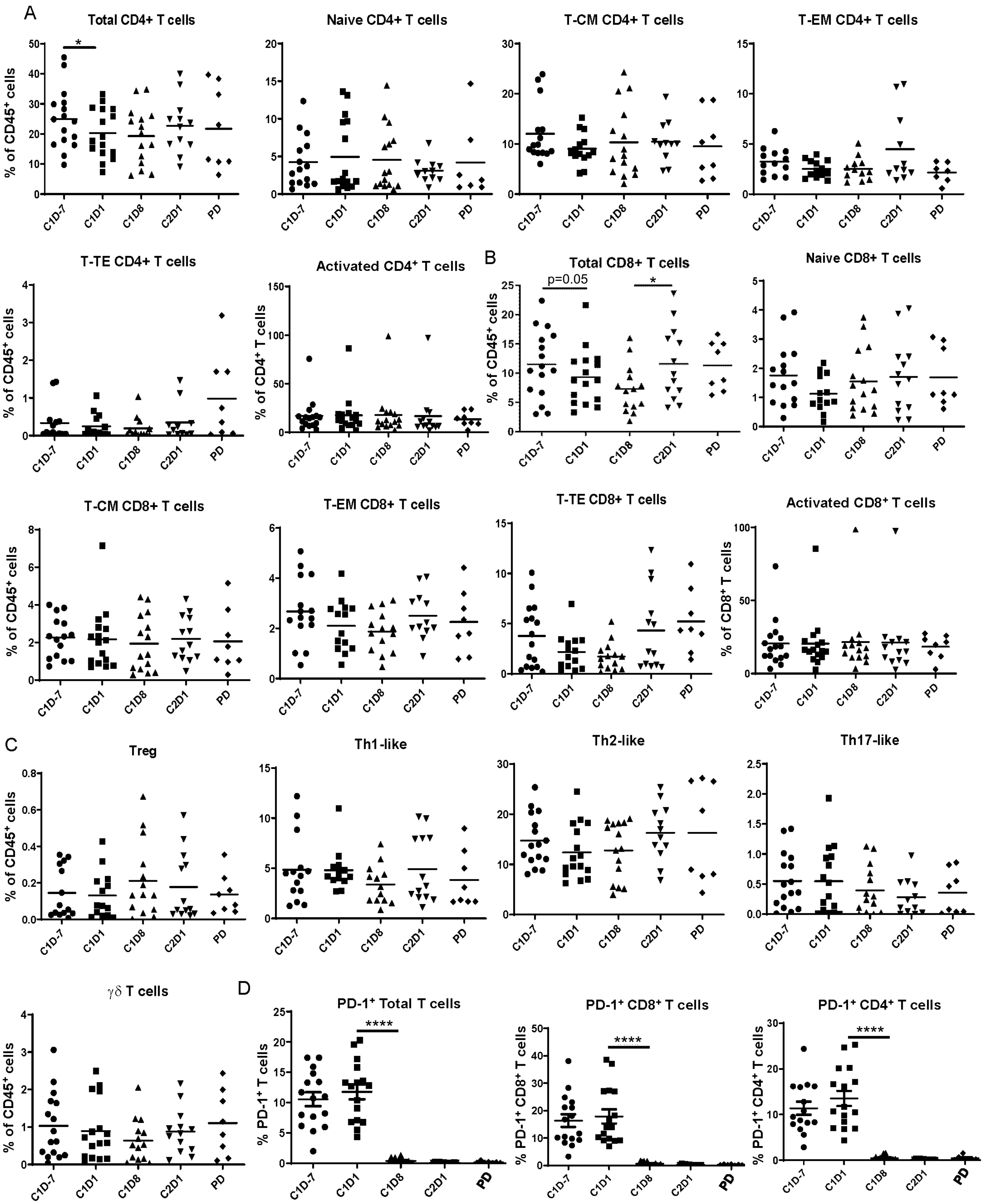

Supplemental Figure 5. Effect of ibrutinib and nivolumab on T cells. (A) Percentages of $CD4^+$ and (B) $CD8^+$ T cells isolated from the peripheral blood of patients (n=16) at the indicated timepoints and at time of disease progression (PD). T cells were differentiated into subsets based

on the expression of CD45RA and CCR7; Naïve T cells ($CD45RA^+$ $CCR7^+$), central memory T cells ($CD45RA^-$ $CCR7^+$), effector memory T cells ($CD45RA^-$ $CCR7^-$), terminal effector memory T cells ($CD45RA^+$ $CCR7^-$) and activated T cells ($CD69^+$). (C) Percentages of regulatory T cells (Treg), Th1, Th2, Th17, and γδ T cell subsets. (D) Percentages of PD-1^+ total, $CD8^+$ and $CD4^+$ T cells. Cell population data was obtained using mass cytometry. Data represent mean ± SEM. Data are analyzed by student's *t*-test (paired), and p-values are adjusted for multiple comparisons using Holm-Bonferroni method, *$p<0.05$, **$p<0.01$, ***$p<0.001$, ****$p<0.0001$.

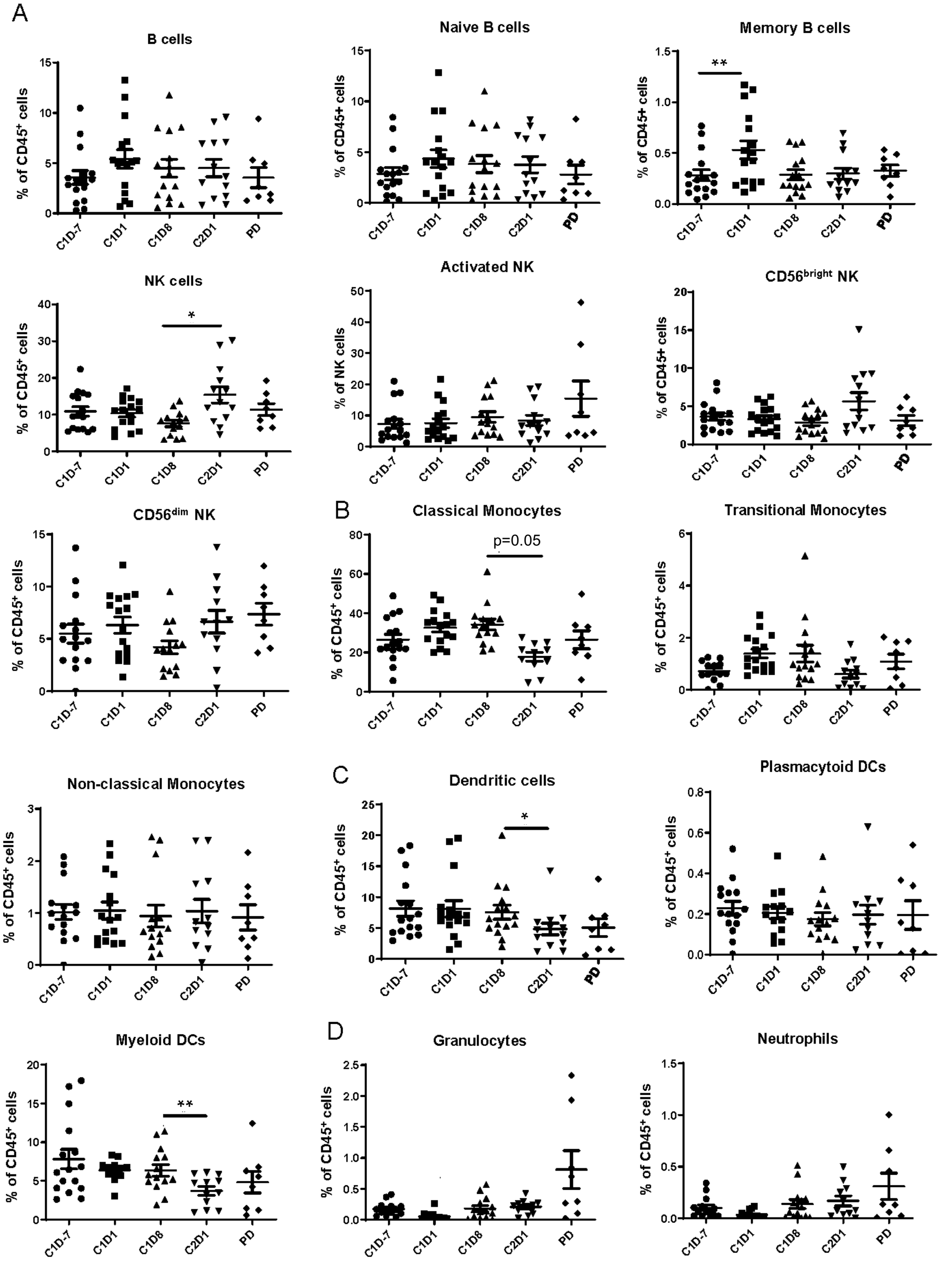

Supplemental Figure 6. Effect of ibrutinib and nivolumab on circulating immune cell subsets. Cell population data obtained using mass cytometry. Percentages of (A) total, naïve and memory B cells, total, activated ($CD69^{+}$), $CD56^{bright}$ and $CD56^{dim}$ NK cells, (B) classical, transitional, and non-classical monocytes, (C) dendritic cells (DCs), plasmacytoid DCs, and myeloid DCs, (D) granulocytes and neutrophils isolated from the peripheral blood of patients at the indicated timepoints and at disease progression (PD). Data represent mean ± SEM (n=16 patients). Data are analyzed by student's *t*-test (paired), and p-values are adjusted for multiple comparisons using Holm-Bonferroni method, *$p<0.05$, **$p<0.01$.

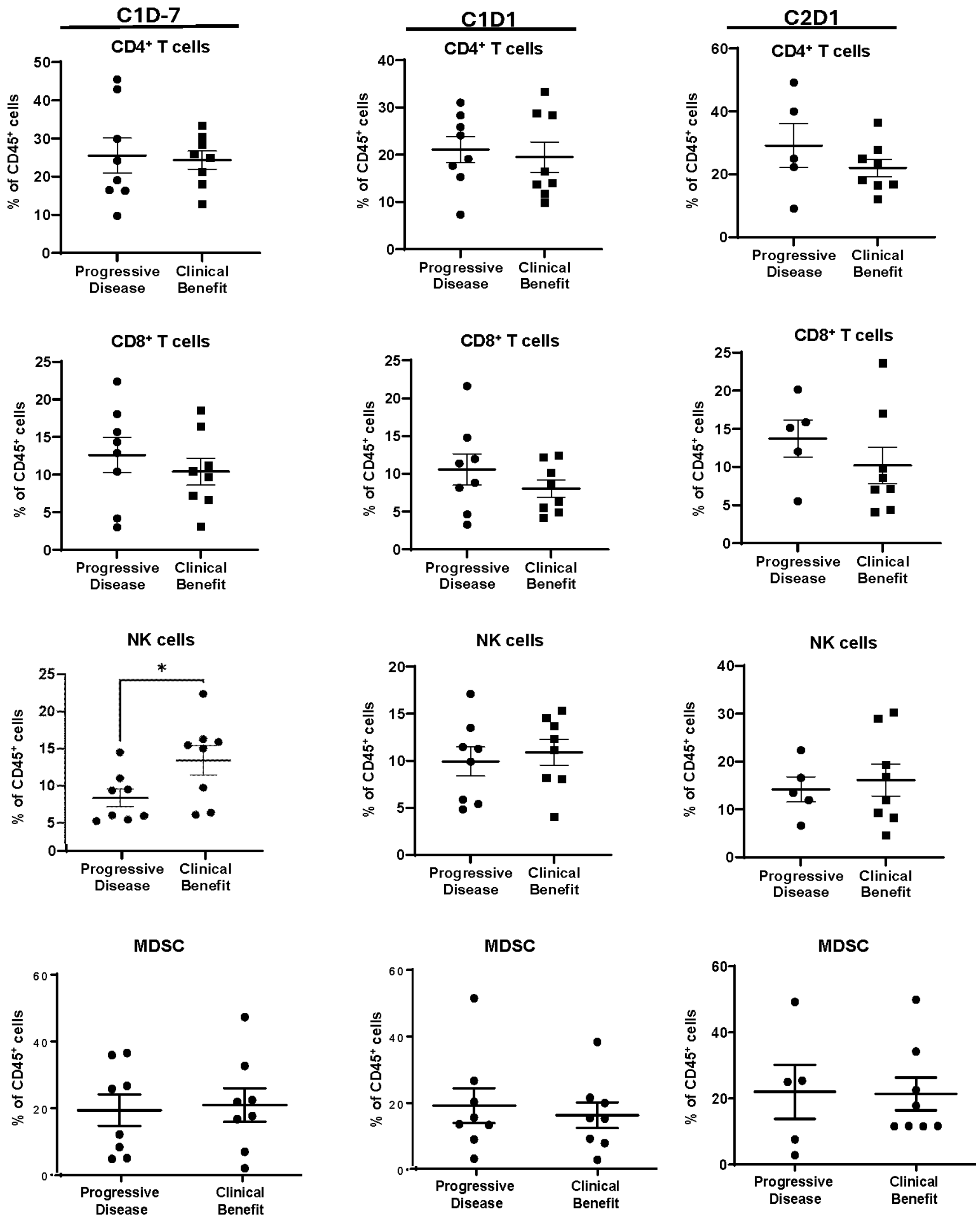

Supplemental Figure 7. Comparison of circulating immune cell populations between progressive disease and clinically benefitting patients. Quantification of major immune cell populations isolated from the peripheral blood of study patients at the indicated timepoints. Cell population data was obtained using mass cytometry. Data are presented as mean normalized intensity ± SE (n=16 patients). Clinical benefit included PR=partial response (n=4) and SD=stable disease (n=4). PD = progressive disease (n=8). Student's *t*-tests (un-paired) were used to compare clinical benefit (PR+SD) and PD at each timepoint for each cell type respectively, *p<0.05.

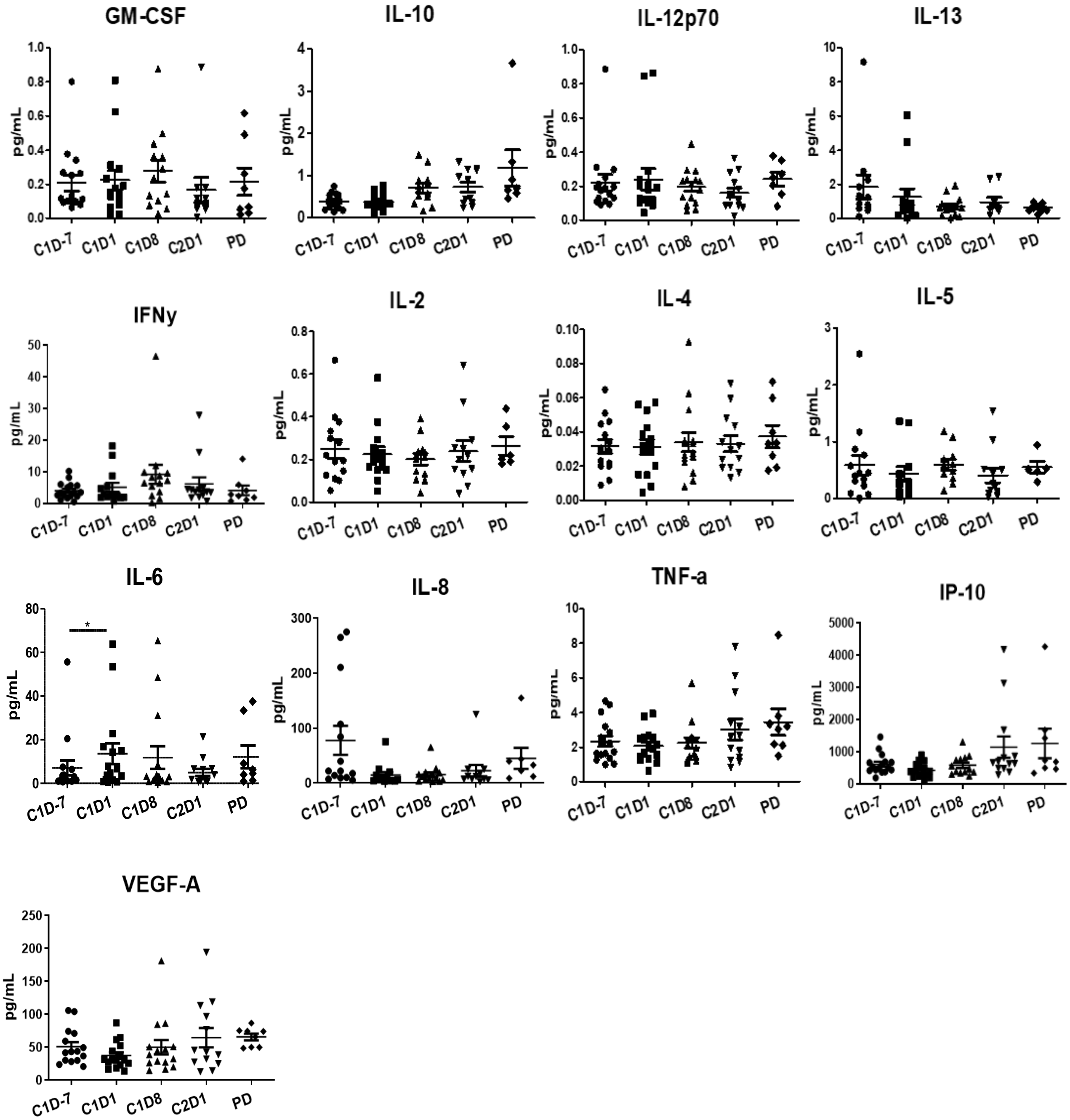

Supplemental Figure 8. Serum levels of cytokines vary over the course of study therapy. Serum levels of GM-CSF, IL-10, IL-12p70, IL-13, IFN-γ, IL-2, IL-4, IL-5, IL-6, IL-8, TNF-α, IP-10 and VEGF-A were measured prior to cycle 1 day -7 (baseline), cycle 1 day 1, cycle 1 day 8, cycle 2 day 1 and at the time of disease progression (PD) using a custom U-PLEX Human Cytokine Panel 20-plex Assay. The assay was performed in duplicate and cytokine levels were measured for all patients and displayed as mean ± SEM. Data are analyzed by student's *t*-test (paired), and p-values are adjusted for multiple comparisons within each biomarker using Holm-Bonferroni method, *$p<0.05$.

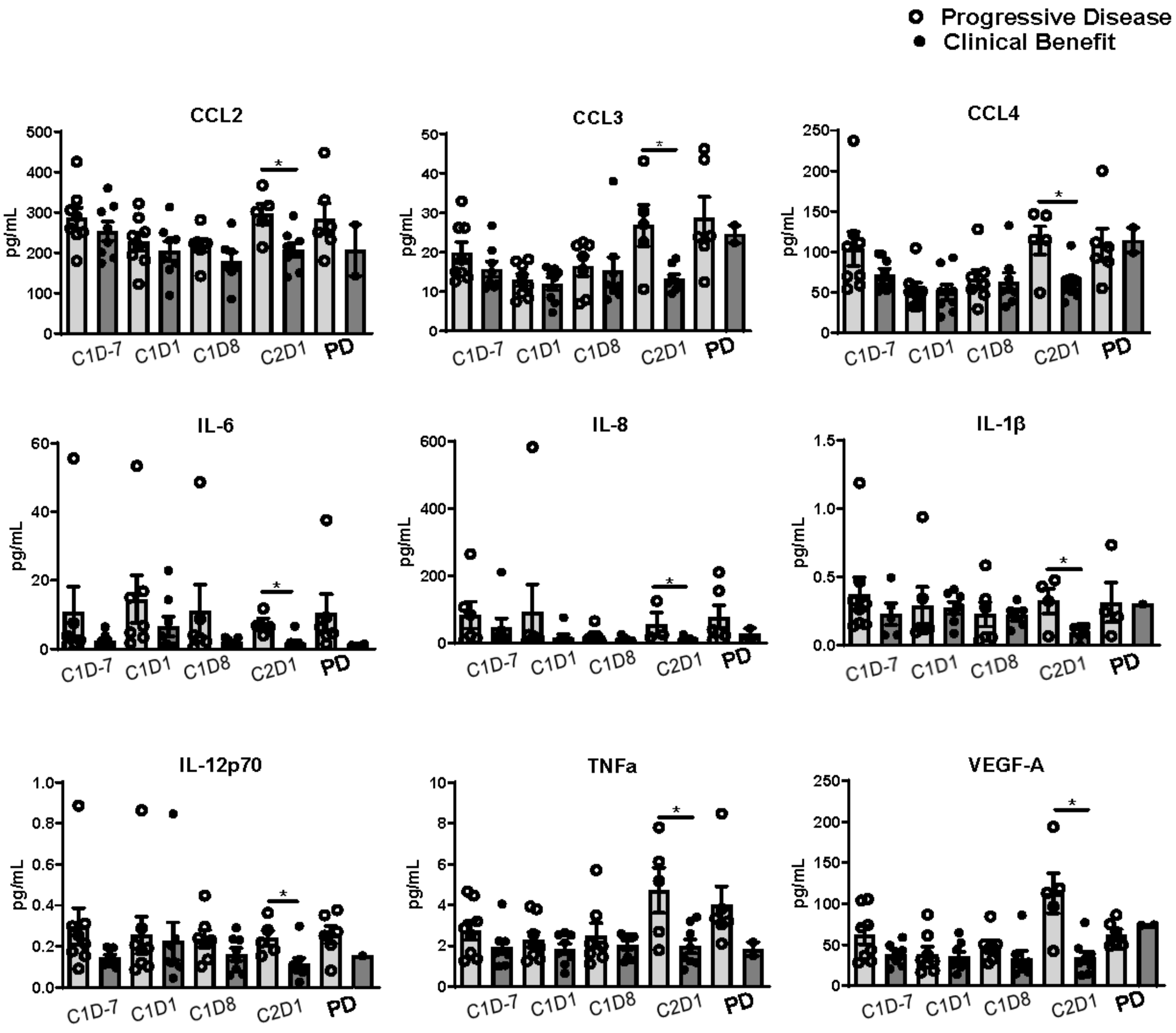

Supplemental Figure 9. Serum levels of CCL2, CCL3, CCL4, IL-6, IL-8, IL-1β, IL-12p70, TNFα and VEGF-A vary by response. Serum levels of 20 cytokines and chemokines were measured at the indicated timepoints and at time of disease progression (PD). Patients were grouped as clinical benefit (partial response and stable disease) or progressive disease. Levels of CCL2, CCL3, CCL4, IL-6, IL-8, IL-1β, IL-12p70, TNFα and VEGF-A at cycle 2, day 1 (C2D1) were significantly decreased in clinically benefitting patients compared to progressive disease. Student's *t*-tests (un-paired) were used to compare clinical benefit (PR+SD) and PD at each timepoint for each cytokine respectively, *p<0.05.

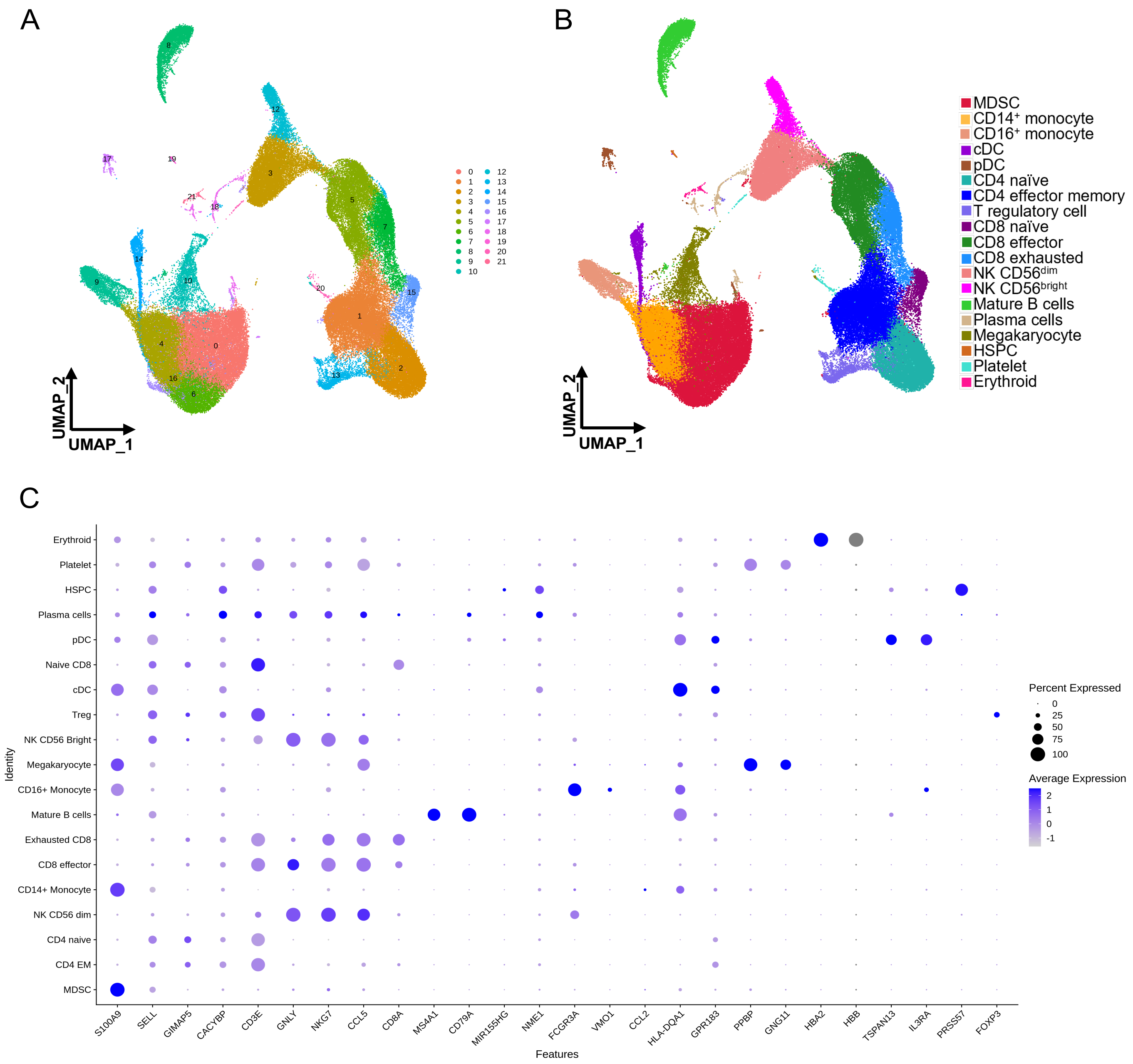

Supplemental Figure 10. Louvain clustering and subcluster identification. (A) Louvain clustering of single-cell RNA-sequencing data from patients (n=16) at C1D-7 and C1D1 (n=30 samples). Individual clusters identified by expression of canonical gene markers are labeled as 0-21. **(B)** Annotated clusters generated using SingleR and manually using PanglaoDB (66). **(C)** Heatmap of canonical gene markers used to verify cluster annotation. Circle sizes are proportional to the percent expression of each marker. Cricle color represents the average expression of each marker with a darker color corresponding to a higher expression level.

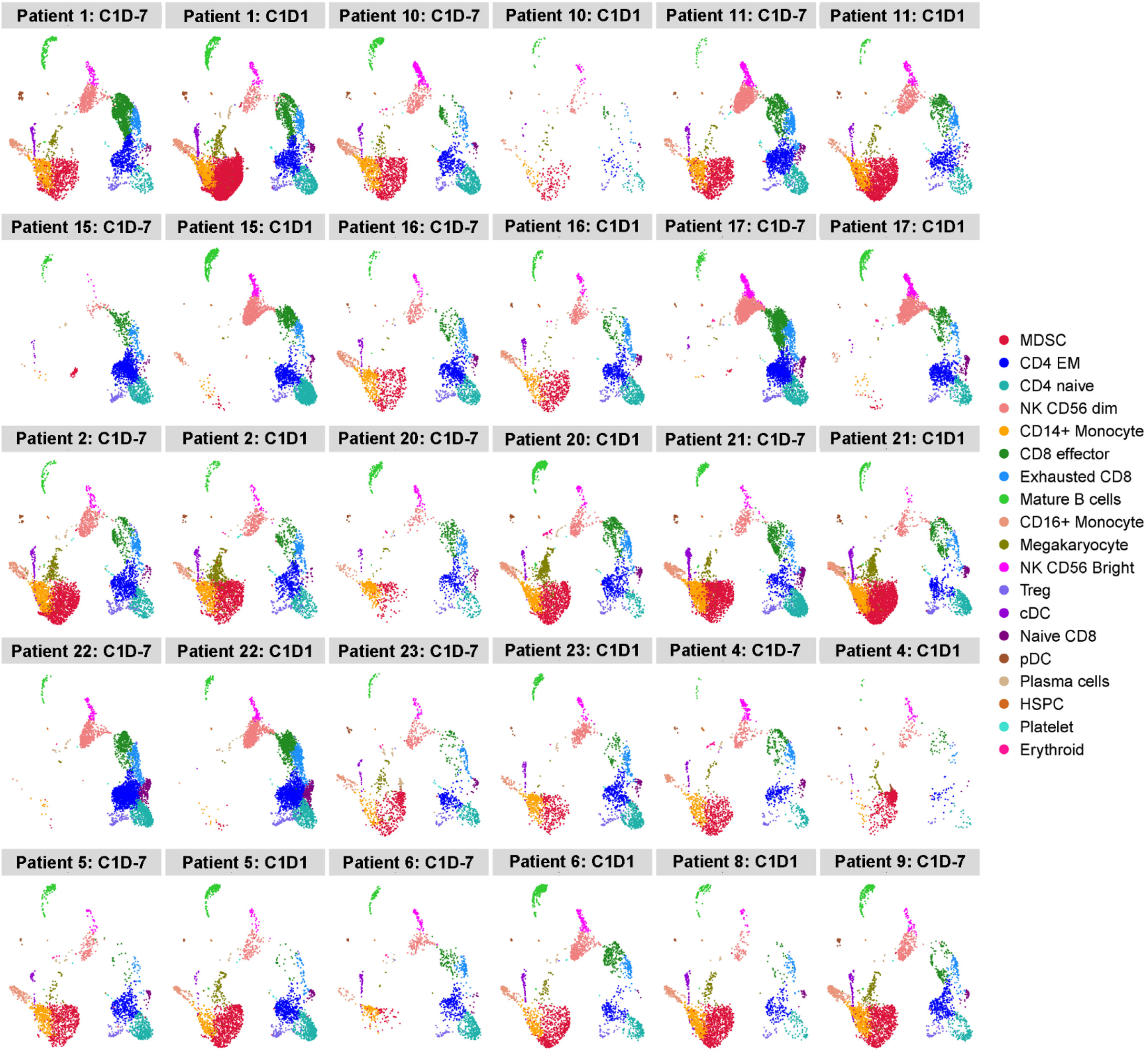

Supplemental Figure 11. Individual patient UMAPs. UMAPs for each patient (n=16) before (C1D-7) and/or after 7 days of ibrutinib therapy (C1D1).

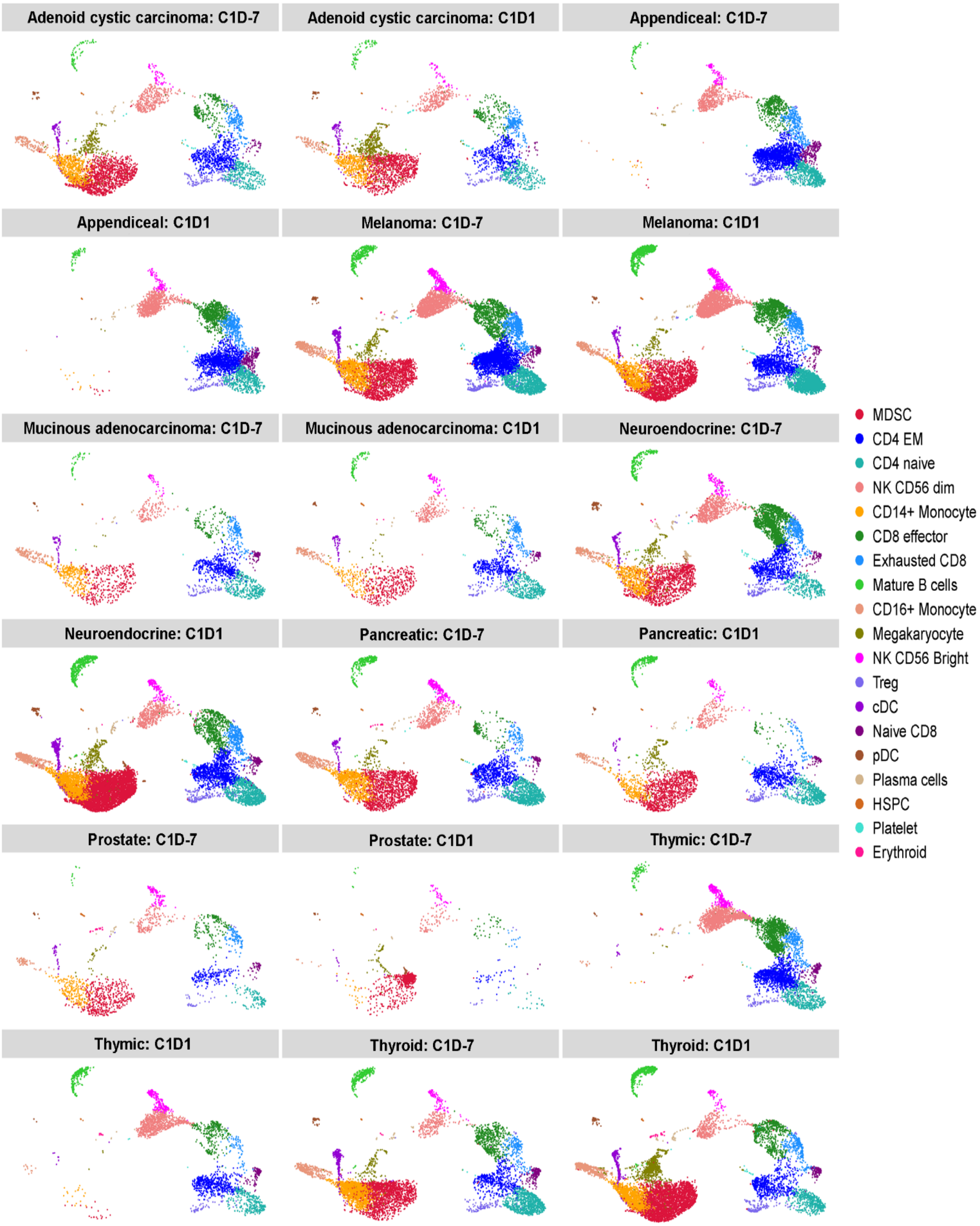

Supplemental Figure 12. Tumor type specific UMAPs. Individual UMAPs for each tumor type including (adenoid cystic carcinoma: n=1, appendiceal: n=1, melanoma: n=4, mucinous adenocarcinoma: n=1, neuroendocrine: n=3, pancreatic: n=2, prostate: n=1, thymic: n=1, thyroid: n=2) before (C1D-7) and after 7 days of ibrutinib therapy (C1D1).

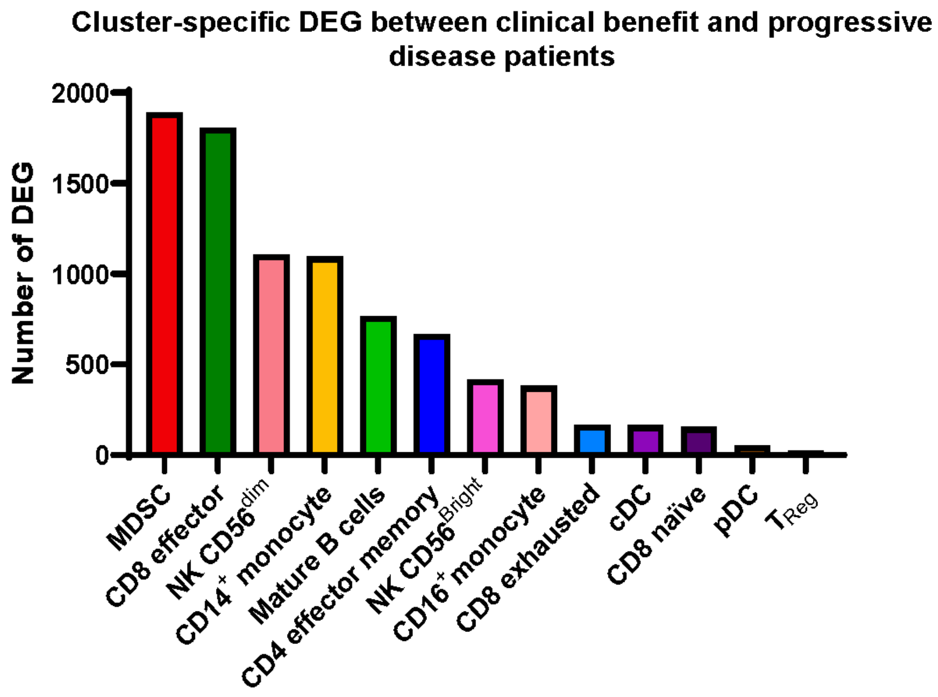

Supplemental Figure 13. Analysis of cluster-specific differentially expressed genes between patient response groups. Gene expression data was calculated based on paired C1D-7 and C1D1 single-cell RNA-seq samples from patients classified as clinical benefit (partial response or stable disease) (n=6 patients, n=12 samples) and patients classified as having progressive disease (n=8 patients, n=16 samples). The number of genes differentially expressed between patients with clinical benefit and patients with progressive disease within individual immune cell clusters were then calculated.

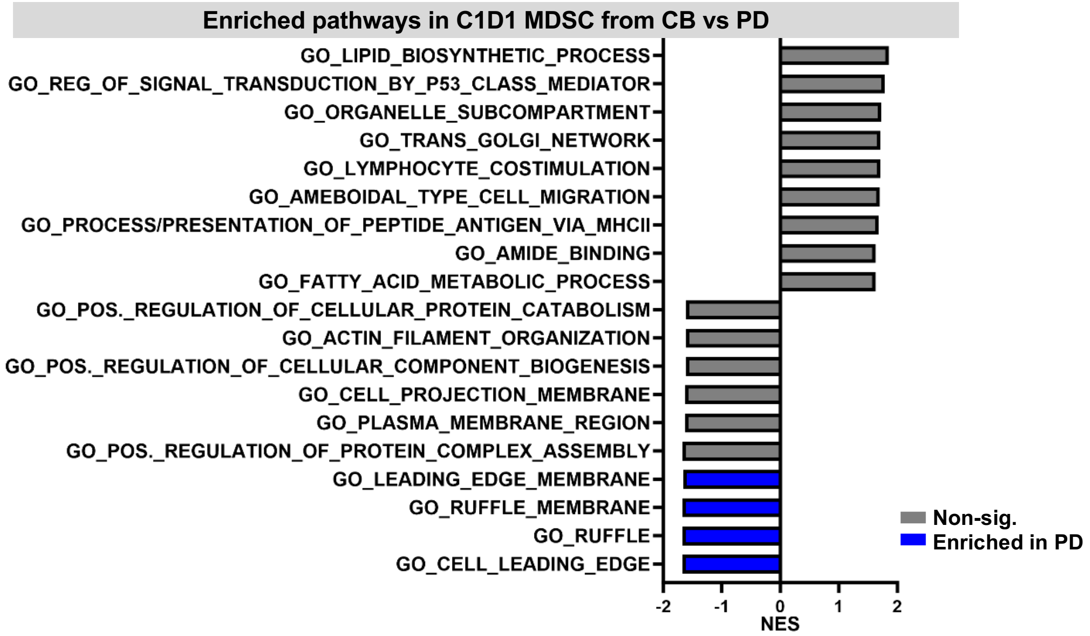

Supplemental Figure 14. Pathway enrichment analysis of ibrutinib-treated MDSC from patients with clinical benefit vs patients with progressive disease. Pathway analysis of the top differentially expressed genes (DEG) in the C1D1 MDSC population in patients with clinical benefit (CB) versus patients with progressive disease (PD). Enrichment is displayed as a normalized enrichment score (NES). Pathways with a Bonferroni-corrected p-value < 0.05 were considered significantly enriched. Pathways significantly enriched in PD patients compared to CB patients are shown in blue. Non-significantly enriched pathways are shown in gray.

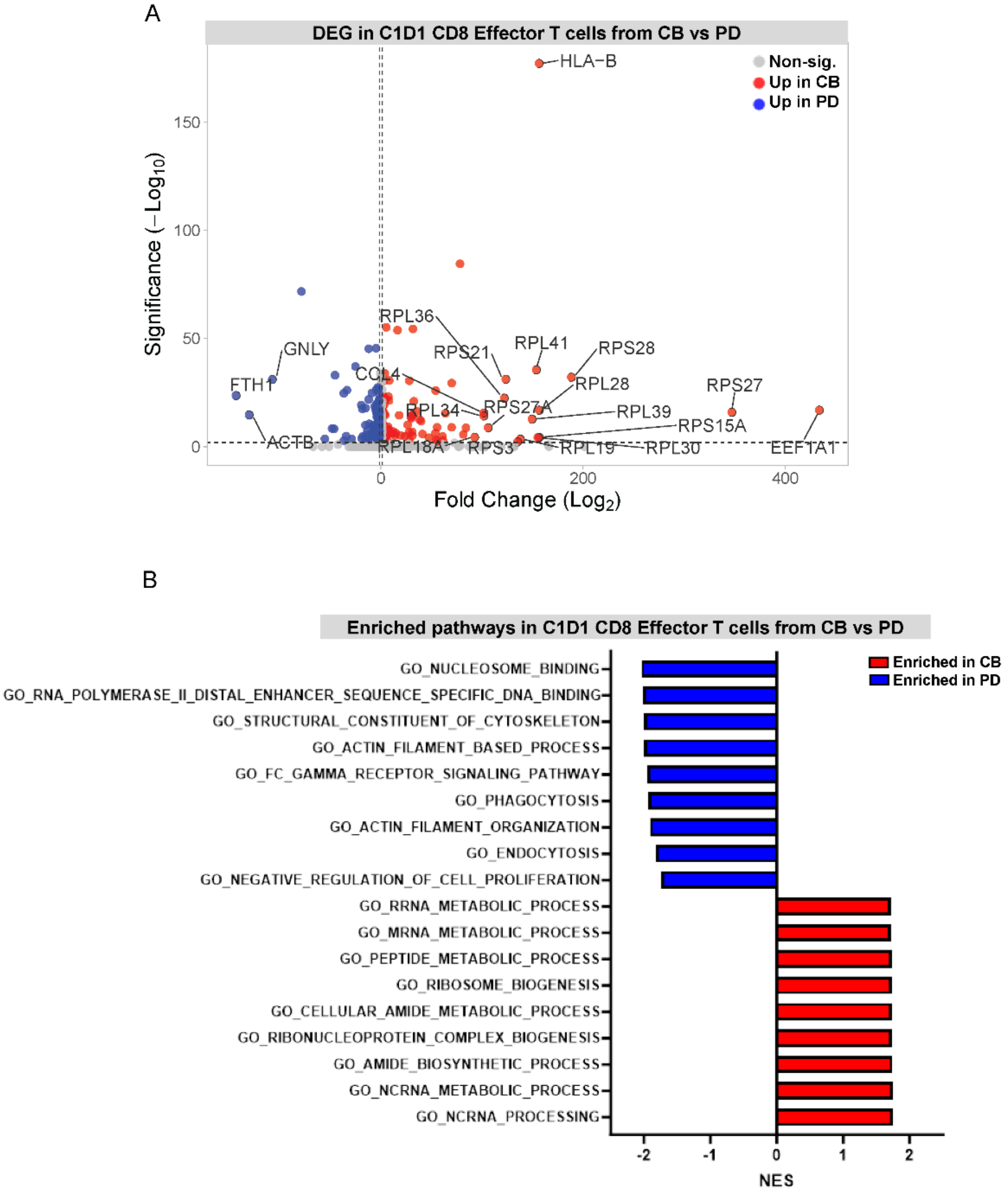

Supplemental Figure 15. Gene expression and pathay enrichment differences in ibrutinib-treated CD8$^+$ effector T cells between patient response groups. (A) Volcano plot of top differentially expressed genes after ibrutinib treatment (C1D1) in the CD8$^+$ effector T cell population from patients with clinical benefit (CB, n=6 patients) versus patients with progressive disease (PD, n=8 patients). Genes downregulated in CD8$^+$ effector T cells from clinical benefit patients relative to CD8$^+$ effector T cells from patients with progressive disease are represented in blue and genes upregulated in CD8$^+$ effector T cells from clinical benefit patients relative to progressive disease patients are represented in red. (x = log2 fold change/ y = -log10(Adjusted P value). (B) Pathway analysis of the top differentially expressed genes in C1D1 CD8$^+$ effector T cells from patients with clinical benefit versus patients with progressive disease. Pathways significantly enriched in clinical benefit patients compared to progressive disease patients are shown in red and pathways significantly enriched in progressive disease patients compared to clinical benefit patients are shown in blue.

Supplemental Table 1. Schedule of sample collection and study schema.

	C1D-7	C1D1	C1D8	C1D15	C2D1	C3+	Progressive Disease
Ibrutinib	X →						
Nivolumab		X		X	X	X	
Peripheral blood collection	X	X	X		X		X

Supplemental Table 2. Demographics and patient characteristics (n=16).

	Total (N=16)
**Age (years)**	
Mean	60.3
Median	60.5
Min, Max	31, 81
SD	13.48
**Gender, *n (%)***	
Male	9 (56)
Female	7 (44)
**Race, *n (%)***	
White	14 (88)
African American	1 (6)
Unknown/declined	1 (6)
**Primary Malignancy, *n (%)***	
Adenoid cystic carcinoma of vulva	1 (6)
Appendiceal mucinous adenocarcinoma	1 (6)
Melanoma	4 (25)
Mucinous adenocarcinoma with gynecologic origin	1 (6)
Neuroendocrine	3 (19)
Pancreatic adenocarcinoma	2 (13)
Prostatic adenocarcinoma	1 (6)
Thymic carcinoma	1 (6)
Thyroid	
Anaplastic	1 (6)
Follicular	1 (6)
**ECOG PS, *n (%)***	
0	8 (50)
1	7 (44)
2	1 (6)
**Prior Systemic Therapy, *n (%)***	
Yes	12 (75)
No	4 (25)
**Number of Prior Lines of Therapy, *n (%)***	
0	5 (31)
1	4 (25)
2	0 (0)
3	3 (19)
>3	4 (25)
**Prior Checkpoint Therapy, *n (%)***	
Yes	0
No	16 (100)

ECOG PS = Eastern Cooperative Oncology Group performance status

Supplemental Table 3. Summary of adverse events associated with ibrutinib and nivolumab combination therapy.

ADVERSE EVENTS	Grade 1-2 N (%)	Grade 3 N (%)	Total (N=16)
**Blood and lymphatic system disorders**			
Anemia	-	2 (12.5)	2 (12.5)
Thrombocytopenia	1 (6.25)	-	1 (6.25)
**Cardiac disorders**			
Atrial fibrillation	1 (6.25)	-	1 (6.25)
Palpitations	1 (6.25)	-	1 (6.25)
Sinus bradycardia	1 (6.25)	-	1 (6.25)
**Gastrointestinal disorders**			
Aspartate aminotransferase increased	1 (6.25)	-	1 (6.25)
Blood bilirubin increased	1 (6.25)	-	1 (6.25)
Diarrhea	1 (6.25)	-	1 (6.25)
Nausea	2 (12.5)	1 (6.25)	3 (19)
Oral mucositis	2 (12.5)	-	2 (12.5)
Vomiting	3 (19)	-	3 (19)
**General, metabolic, and psychiatric disorders**			
Agitation	1 (6.25)	-	1 (6.25)
Anorexia	5 (31)	-	5 (31)
Fatigue	5 (31)	-	5 (31)
Fever	1 (6.25)	-	1 (6.25)
Hyperglycemia	1 (6.25)	-	1 (6.25)
Hypokalemia	1 (6.25)	-	1 (6.25)
Insomnia	1 (6.25)	-	1 (6.25)
**Renal disorders**			
Creatinine increased	1 (6.25)	-	1 (6.25)
**Respiratory disorders**			
Dyspnea	1 (6.25)	-	1 (6.25)
Hemoptysis	1 (6.25)	-	1 (6.25)
Lung infection	-	1 (6.25)	1 (6.25)
Pleural Effusion	1 (6.25)	-	1 (6.25)
**Skin and musculoskeletal disorders**			
Maculo-papular rash	2 (12.5)	2 (12.5)	4 (25)
Myalgia	2 (12.5)	-	2 (12.5)
Non-specific skin disorders	3 (19)	-	3 (19)
Papulo-pustular rash	1 (6.25)	-	1 (6.25)
Pruritus	2 (12.5)	-	2 (12.5)

Supplemental Table 4. Summary of best overall tumor response.

Best Overall Response, n (%)	Total (N=16)
Complete Response	0
Partial Response	4 (25%)
Stable Disease	4 (25%)
Progressive Disease	8 (50%)

Supplemental Table 5. Maxpar direct immune profiling assay 37-marker panel with clones and heavy metals.

CyTOF Profiling Panel			
**Target**	**Clone**	**Metal**	**Source**
CD45	HI30	89Y	Fluidigm
CD196/CCR6	G034E3	141Pr	Fluidigm
CD15	W6D3	142Nd	Fluidigm
CD123	6H6	143Nd	Fluidigm
CD19	HIB19	144Nd	Fluidigm
CD4	RPA-T4	145Nd	Fluidigm
CD8a	RPA-T8	146Nd	Fluidigm
CD11c	Bu15	147Sm	Fluidigm
CD16	3G8	148Nd	Fluidigm
CD45RO	UCHL1	149Sm	Fluidigm
CD45RA	HI100	150Nd	Fluidigm
CD161	HP-3G10	151Eu	Fluidigm
CD194/CCR4	L291H4	152Sm	Fluidigm
CD25	BC96	153Eu	Fluidigm
CD27	O323	154Sm	Fluidigm
CD57	HCD57	155Gd	Fluidigm
CD183/CXCR3	G025H7	156Gd	Fluidigm
CD185/CXCR5	J252D4	158Gd	Fluidigm
CD69	FN50	159Tb	Fluidigm
CD28	CD28.2	160Gd	Fluidigm
CD38	HB-7	161Dy	Fluidigm
CD73	AD2	162Dy	Fluidigm
CD56/NCAM	NCAM16.2	163Dy	Fluidigm
TCRgd	B1	164Dy	Fluidigm
CD33	WM53	165Ho	Fluidigm
CD294	BM16	166Er	Fluidigm
CD197/CCR7	G043H7	167Er	Fluidigm
CD14	63D3	168Er	Fluidigm
CD39	A1	169Tm	Fluidigm
CD3	UCHT1	170Er	Fluidigm
CD20	2H7	171Yb	Fluidigm
CD66b	G10F5	172Yb	Fluidigm
HLA-DR	LN3	173Yb	Fluidigm
IgD	IA6-2	174Yb	Fluidigm
CD279/PD-1	EH12.2H7	175Lu	Fluidigm
CD127	A019D5	176Yb	Fluidigm
CD11b	ICRF44	209Bi	Fluidigm
Live/dead intercalator	N/A	103Rh	Fluidigm

Supplemental Table 6. Immune cell populations and model definitions.

Index	Populations	Model phenotypes
1	Lymphocytes	CD45+
2	CD3 T cells	CD45+ CD3+ CD14- CD66b- CD20- CD56-
3	CD8 T cells	CD45+ CD3+ CD14- CD66b- CD20- CD56- TCRgd- CD4- CD8+
4	CD8 naïve	CD45+ CD3+ CD14- CD66b- CD20- CD56- TCRgd- CD4- CD8+ CCR7+ CD45RA+
5	CD8 central memory	CD45+ CD3+ CD14- CD66b- CD20- CD56- TCRgd- CD4- CD8+ CCR7+ CD45RA-
6	CD8 effector memory	CD45+ CD3+ CD14- CD66b- CD20- CD56- TCRgd- CD4- CD8+ CCR7- CD45RA-
7	CD8 terminal effector	CD45+ CD3+ CD14- CD66b- CD20- CD56- TCRgd- CD4- CD8+ CCR7- CD45RA+
8	CD4 T cells	CD45+ CD3+ CD14- CD66b- CD20- CD56- TCRgd- CD4+ CD8-
9	CD4 naïve	CD45+ CD3+ CD14- CD66b- CD20- CD56- TCRgd- CD4+ CD8- CCR7+ CD45RA+
10	CD4 central memory	CD45+ CD3+ CD14- CD66b- CD20- CD56- TCRgd- CD4+ CD8- CCR7+ CD45RA-
11	CD4 effector memory	CD45+ CD3+ CD14- CD66b- CD20- CD56- TCRgd- CD4+ CD8- CCR7- CD45RA-
12	CD4 terminal effector	CD45+ CD3+ CD14- CD66b- CD20- CD56- TCRgd- CD4+ CD8- CCR7- CD45RA+
13	Tregs	CD45+ CD3+ CD14- CD66b- CD20- CD56- TCRgd- CD4+ CD8- CD25+ CD127-
14	Th1-like	CD45+ CD3+ CD14- CD66b- CD20- CD56- TCRgd- CD4+ CD8- CXCR5- CXCR3+ CCR6-
15	Th2-like	CD45+ CD3+ CD14- CD66b- CD20- CD56- TCRgd- CD4+ CD8- CXCR5- CXCR3- CCR6-
16	Th17-like	CD45+ CD3+ CD14- CD66b- CD20- CD56- TCRgd- CD4+ CD8- CXCR5- CXCR3- CCR6+
17	γδ T cells	CD45+ CD3+ CD14- CD20- TCRgd+
18	B cells	CD45+ CD3- CD14- CD16- CD161- CD19+ CD20+
19	B naïve	CD45+ CD3- CD14- CD16- CD161- CD19+ CD20+ CD27- IgD+
20	B memory	CD45+ CD3- CD14- CD16- CD161- CD19+ CD20+ CD27+ IgD-
21	NK cells	CD45+ CD3- CD14- CD19- CD20- CD123- CD56+
22	Activated NK cells	CD45+ CD3- CD14- CD19- CD20- CD123- CD56+ CD69+
23	$CD56^{bright}$ NK	CD45+ CD3- CD14- CD19- CD20- CD123- CD56++ CD16-
24	$CD56^{dim}$ NK	CD45+ CD3- CD14- CD19- CD20- CD123- CD56+ CD16+
25	Classical monocytes	CD45+ CD3- CD19- CD20- CD56- CD66b-CD14+ CD16-
26	Transitional monocytes	CD45+ CD3- CD19- CD20- CD56- CD66b-CD14+ CD16+
27	Non-classical monocytes	CD45+ CD3- CD19- CD20- CD56- CD66b-CD14- CD16+
28	DCs	CD45+ CD3- CD19- CD20- CD56- HLADR+ CD16-
29	mDCs	CD45+ CD3- CD19- CD20- CD56- HLADR+ CD16- CD123- CD11c+
30	pDCs	CD45+ CD3- CD19- CD20- CD56- HLADR+ CD16- CD123+ CD11c-
31	Granulocytes	CD45+ CD3- CD66b+
32	Neutrophils	CD45+ CD3- CD19- CD20- CD56- HLADR- CD16+ CD66b+
33	MDSC	CD45+ CD3- CD19- CD20- CD56- CD11b+ CD33+ HLADR-/lo
34	M-MDSC	CD45+ CD3- CD19- CD20- CD56- CD11b+ CD33+ HLADR-/lo CD14+ CD66b-
35	G-MDSC	CD45+ CD3- CD19- CD20- CD56- CD11b+ CD33+ HLADR-/lo CD14- CD66b+

Supplemental Table 7. Top 15 up- and downregulated genes after 7 days of ibrutinib therapy in MDSC from all patients and patients according to clinical response.

C1D1 vs C1D-7 MDSC

All Patients

Upregulated with Ibrutinib		
Gene	avg_log2FC	p_val_adj
S100A9	271.96	3.60E-27
S100A12	98.84	7.22E-07
MT-CO3	83.82	8.91E-06
MT-CO1	65.66	1.27E-05
MT-CYB	51.05	1.66E-02
RPS23	32.92	4.58E-02
NCF1	32.43	1.53E-03
IFITM2	29.56	2.11E-19
HMGN2	28.81	7.40E-07
RPL11	25.04	1.65E-32
RPL12	18.16	1.74E-21
RPL30	18.05	2.17E-33
RPLP1	16.61	5.13E-05
RPS7	16.61	1.82E-09
LGALS2	16.60	2.99E-81

Downregulated with Ibrutinib		
Gene	avg_log2FC	p_val_adj
FTH1	-309.44	0.00E+00
MALAT1	-140.65	2.12E-16
TIMP1	-114.23	2.47E-110
B2M	-88.71	2.24E-25
EEF1A1	-82.94	1.22E-07
RPL10	-80.05	7.90E-28
RPS27	-77.17	1.07E-06
FABP5	-61.30	3.05E-14
AREG	-60.15	9.67E-45
RPS12	-59.67	3.55E-10
RPS15A	-58.84	4.45E-17
SRGN	-55.32	3.28E-194
G0S2	-54.20	0.00E+00
RPL34	-49.69	3.99E-24
GNLY	-46.87	9.94E-05

Clinical Benefit

Upregulated with Ibrutinib		
Gene	avg_log2FC	p_val_adj
S100A8	404.99	2.39E-20
S100A9	125.11	2.00E-02
S100A12	120.78	4.07E-07
TMSB10	86.61	6.80E-05
GNLY	68.83	1.15E-12
ACTB	61.81	1.24E-11
B2M	61.18	1.05E-07
LGALS1	53.32	7.66E-12
NCF1	39.97	6.86E-14
S100A6	35.64	5.82E-19
RETN	22.12	4.72E-06
CCL3	14.52	2.38E-30
VCAN	14.09	1.71E-05
IL32	12.58	7.33E-09
CCL4	12.55	3.38E-06

Downregulated with Ibrutinib		
Gene	avg_log2FC	p_val_adj
RPS27	-140.35	0.00E+00
RPL39	-104.09	2.03E-16
RPL13	-98.51	4.00E-02
RPL41	-88.4	1.19E-28
RPL32	-76.84	2.00E-02
RPS28	-69.66	2.86E-21
RPS21	-65.32	1.74E-20
RPL37	-64.08	8.39E-11
RPLP1	-59.56	4.68E-09
RPL7A	-59.46	4.00E-02
FTL	-55.23	2.03E-17
RPL34	-53.76	3.00E-02
RPL18	-52.62	2.15E-06
RPL8	-49.76	2.00E-02
RPS15	-48.02	3.50E-11

Progressive Disease

Upregulated with Ibrutinib		
Gene	avg_log2FC	p_val_adj
S100A9	271.69	4.77E-23
TMSB10	115.69	2.30E-14
S100A12	98.57	1.60E-04
MT-CO1	88.47	1.22E-14
ACTG1	75.46	3.94E-11
RPL39	73.58	2.06E-101
RPS27	66.82	3.61E-66
RPLP1	62.32	2.14E-58
RPL41	50.86	1.38E-33
RPS28	49.51	1.07E-89
RPL30	37.97	2.96E-94
RPS23	33.64	1.08E-30
RPS18	33.61	1.58E-23
RPS12	30.94	1.60E-48
IFITM2	29.29	1.39E-04

Downregulated with Ibrutinib		
Gene	avg_log2FC	p_val_adj
MALAT1	-347.23	4.69E-11
FTH1	-309.72	2.97E-292
TIMP1	-127.86	8.84E-200
GNLY	-88.79	5.04E-23
CCL2	-66.55	1.26E-137
EEF1A1	-65.9	3.41E-29
CCL7	-61.57	2.26E-115
RPL13	-60.13	9.51E-31
CTSL	-58.7	8.05E-298
LYZ	-58.51	1.49E-20
VIM	-55.65	1.87E-32
G0S2	-54.36	0.00E+00
SRGN	-50.59	2.92E-214
CXCL2	-48.07	1.80E-49
IL32	-44.05	1.78E-11

Supplemental Table 8. Top 15 up- and downregulated genes after 7 days of ibrutinib therapy in CD8$^+$ effector T cells from all patients and patients according to clinical response.

C1D1 vs C1D-7 CD8 Effector T cells

All Patients

Upregulated with Ibrutinib		
Gene	avg_log2FC	p_val_adj
MALAT1	167.91	5.44E-03
ACTB	47.98	1.98E-33
RPL38	43.76	9.37E-03
ACTG1	41.95	3.49E-15
CCL5	41.60	1.17E-26
RPS2	39.31	2.86E-36
LTB	36.63	8.50E-03
RPL32	27.95	1.93E-04
RPL13	27.79	2.94E-09
IL32	20.30	4.27E-18
OASL	17.96	3.15E-02
VIM	17.87	1.59E-02
RPL29	12.45	3.83E-05
BTG1	12.12	4.22E-03
TXNIP	11.88	8.27E-92

Downregulated with Ibrutinib		
Gene	avg_log2FC	p_val_adj
GNLY	-368.77	2.03E-11
RPS26	-228.82	2.55E-50
MT-CO3	-228.79	1.51E-28
MT-CO2	-176.44	4.33E-42
RPL11	-136.50	9.35E-05
RPL12	-97.54	1.22E-06
RPL8	-93.17	1.74E-08
RPL28	-81.67	4.96E-02
MT-CYB	-75.90	3.03E-32
HLA-B	-61.90	2.16E-24
MT-ND1	-49.06	2.50E-10
MT-ATP8	-44.16	3.29E-29
RPS12	-44.16	2.71E-02
B2M	-41.27	9.62E-14
PTMA	-32.62	6.14E-27

Clinical Benefit

Upregulated with Ibrutinib		
Gene	avg_log2FC	p_val_adj
CCL4	129.11	7.46E-03
ACTB	50.66	1.47E-50
RPL38	47.98	2.86E-15
ACTG1	26.87	1.82E-16
TMSB10	17.84	3.03E-23
PFN1	15.23	1.58E-43
RPS29	9.46	1.19E-12
RPL27A	8.61	8.29E-03
TRDV3	8.22	1.08E-17
MIF	8.06	3.43E-15
CCL5	8.03	6.07E-52
ATP5F1E	7.85	9.70E-11
GADD45B	7.69	3.54E-02
TUBA1B	7.05	2.85E-10
TRDV1	6.24	4.72E-04

Downregulated with Ibrutinib		
Gene	avg_log2FC	p_val_adj
MT-CO3	-234.18	1.21E-20
RPS26	-228.40	1.03E-05
MT-CO2	-199.55	1.02E-02
FTH1	-128.68	1.15E-33
RPL12	-123.09	1.65E-02
B2M	-85.57	1.61E-50
RPS18	-84.57	4.22E-06
PTMA	-55.19	6.07E-22
MT-ATP8	-43.74	2.95E-18
HLA-A	-37.96	6.44E-04
LMNA	-32.00	3.21E-13
LEPROTL1	-26.80	7.30E-24
AHNAK	-25.11	7.49E-07
MT2A	-23.09	1.28E-04
CD74	-23.09	6.02E-02

Progressive Disease

Upregulated with Ibrutinib		
Gene	avg_log2FC	p_val_adj
MALAT1	193.58	4.50E-07
VIM	78.25	8.40E-13
TMSB4X	53.62	1.07E-05
RPS2	39.03	4.51E-17
RPL13	30.56	8.28E-04
ANXA1	30.55	5.76E-07
RPL41	26.23	1.92E-02
EEF1A1	20.46	1.20E-03
IL32	19.99	1.98E-13
RPS15	14.24	3.15E-02
TXNIP	14.22	1.34E-09
MT-ND2	13.61	1.61E-03
RACK1	13.00	4.78E-02
LTB	11.80	6.24E-04
JUN	10.18	1.44E-08

Downregulated with Ibrutinib		
Gene	avg_log2FC	p_val_adj
GNLY	-89.18	7.66E-33
MT-CO2	-58.44	6.97E-40
MT-CO1	-53.09	7.90E-12
B2M	-41.57	5.10E-13
MT-CYB	-39.68	5.33E-41
MT-CO3	-34.12	1.69E-05
CCL3	-25.76	2.77E-04
JUNB	-24.26	4.78E-32
S100A4	-21.37	6.49E-40
HLA-DRB1	-21.37	6.06E-06
RPS26	-18.58	5.60E-34
CD74	-18.49	9.67E-04
CRIP1	-18.30	1.07E-06
HLA-C	-17.16	4.08E-72
FGFBP2	-15.33	7.73E-09
